# To Probe Full and Partial Activation of Human Peroxisome Proliferator-Activated Receptors by Pan-Agonist Chiglitazar Using Molecular Dynamics Simulations

**DOI:** 10.1155/2020/5314187

**Published:** 2020-04-01

**Authors:** Holli-Joi Sullivan, Xiaoyan Wang, Shaina Nogle, Siyan Liao, Chun Wu

**Affiliations:** ^1^College of Science and Mathematics, Rowan University, Glassboro, NJ 08028, USA; ^2^School of Radiology, Taishan Medical University, Tai'an, Shandong 271016, China; ^3^Medical School, Southeast University, Nanjing 210009, China; ^4^Key Laboratory of Molecular Target & Clinical Pharmacology, School of Pharmaceutical Sciences, Guangzhou Medical University, Guangzhou 511436, China

## Abstract

Chiglitazar is a promising new-generation insulin sensitizer with low reverse effects for the treatment of type II diabetes mellitus (T2DM) and has shown activity as a nonselective pan-agonist to the human peroxisome proliferator-activated receptors (PPARs) (i.e., full activation of PPAR*γ* and a partial activation of PPAR*α* and PPAR*β*/*δ*). Yet, it has no high-resolution complex structure with PPARs and its detailed interactions and activation mechanism remain unclear. In this study, we docked chiglitazar into three experimentally resolved crystal structures of hPPAR subtypes, PPAR*α*, PPAR*β*/*δ*, and PPAR*γ*, followed by 3 *μ*s molecular dynamics simulations for each system. Our MM-GBSA binding energy calculation revealed that chiglitazar most favorably bound to hPPAR*γ* (-144.6 kcal/mol), followed by hPPAR*α* (-138.0 kcal/mol) and hPPAR*β* (-135.9 kcal/mol), and the order is consistent with the experimental data. Through the decomposition of the MM-GBSA binding energy by residue and the use of two-dimensional interaction diagrams, key residues involved in the binding of chiglitazar were identified and characterized for each complex system. Additionally, our detailed dynamics analyses support that the conformation and dynamics of helix 12 play a critical role in determining the activities of the different types of ligands (e.g., full agonist vs. partial agonist). Rather than being bent fully in the direction of the agonist versus antagonist conformation, a partial agonist can adopt a more linear conformation and have a lower degree of flexibility. Our finding may aid in further development of this new generation of medication.

## 1. Introduction

In the year 1999, the World Health Organization estimated that by 2025 roughly 300 million people would be suffering from diabetes. However, in 2014, the World Health Organization reported 422 million people suffering from diabetes worldwide, surpassing the estimate by a shocking 122 million people with 11 years to spare. This statistic highlights the ongoing and crucial need for an effective treatment for type II diabetes mellitus (T2DM) [1–3].

Human peroxisome proliferator-activated receptors (PPARs) belong to a subfamily of nuclear hormone receptors that act as ligand-activated transcription factors to regulate a variety of biological processes including glucose metabolism, lipoprotein metabolism, and immune response [4–6]. The ligand-binding domain (LBD) of PPARs forms a heterodimer with the retinoid X receptor (RXR) and binds specific DNA sequences in the regulatory region of target genes to modulate their transcription ([Supplementary-material supplementary-material-1]). Upon ligand binding, conformational changes occur to the PPAR LBD which promotes the recruitment of coactivators such as nuclear receptor coactivator 2 (NCOA2). However, the exact mechanism by which full activation and partial activation occur at the PPAR LBD remains to be fully understood, despite being well studied in the past. A common conception of PPAR full agonists is that the activation mechanism primarily occurs through the stabilization of helix 12 [7] in the activation function 2 (AF-2) region. However, a number of studies show that for both full and partial agonists the activation of the receptor is not solely dependent on the stabilization of helix 12 but that interactions with helices 3, 4, 6, 7, and 11 and the beta region also play a role [8–13]. It has also been shown that the agonists of PPAR can adopt multiple binding poses [9, 14] suggesting that a one true understood mechanism for all PPAR agonists is not feasible and that a detailed binding mode is needed to fully understand the unique activation mechanism of the receptor.

**Figure 1:**
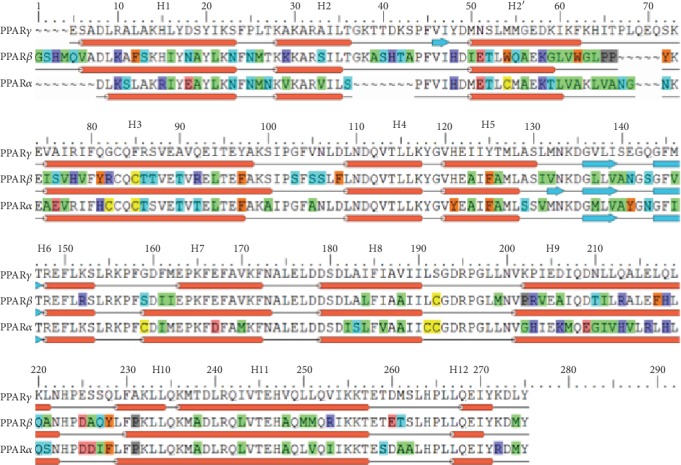
Sequence alignment of PPAR*α* (PDB ID: 3VI8), PPAR*β* (PDB ID: 3TKM), and PPAR*γ* (PDB ID: 2PRG): 202 (alpha), 174 (beta/delta), and 238 (gamma). The sequences were aligned using PPAR*γ* as a reference, and each residue that differs is colored based on side-chain chemistry. See Materials and Methods for reference to colors.

The hPPARs are divided into three distinct subtypes: PPAR*α* (NR1C1), PPAR*β*/*δ* (NR2C2), and PPAR*γ* (NR3C3) (Figure 1), each of which are discrete in terms of expression and biological function. PPAR*α* plays an important role in lipid and glucose metabolism [6, 15–20], PPAR*β*/*δ* is integral in energy metabolism [21], and PPAR*γ* has a variety of implications in adipocyte differentiation and sensitivity, cell cycle regulation, inflammation, and even immune responses [6, 15–18, 20, 22–24]. Thiazolidinediones (TZDs) rosiglitazone (Avandia) and pioglitazone (Actos) are selective full agonists to the PPAR*γ* receptor and were once a common method for T2DM treatment. TZDs act as insulin sensitizers that improve glycemic control but have now become “Nonformulary oral options that are nonpreferred but can be considered in patients at high hypoglycemia risk where cost is an issue” because studies have linked these drugs to hepatotoxicity, increased risk for cardiovascular failure, myocardial infarction, increased risk for bladder cancer, and body weight gain [6, 25–32]. Despite the adverse effects of current medications, development of new PPAR agonists are still of great interest because of the unique and promising feature of this class of drug, including the ability to directly target insulin resistance and provide a more durable glycemic (HbA1c) control when compared to other antidiabetic medications [33]. In an attempt to reduce the reverse effects, alternative approaches were considered to target the PPAR receptors including partial PPAR*γ* agonists [34–44], multitargeted cooperative PPAR*α*/*γ* dual agonists [28, 31, 40, 45–58], and PPAR*α*/*β*/*γ* pan-agonists [30, 35, 59–65].

**Figure 2:**
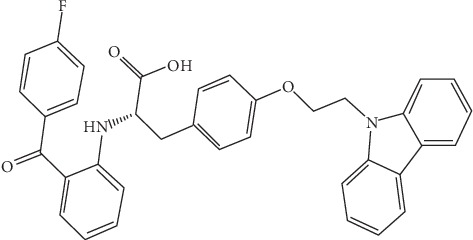
Chemical structure of chiglitazar.

Chiglitazar (Figure 2), discovered and synthesized by Shenzhen Chipscreen Biosciences Ltd., has recently completed phase III clinical trials in China. Chiglitazar is a non-TZD insulin sensitizer and is described as a nonselective pan-agonist to the three PPAR receptor subtypes shown to act on the PPAR*α*, PPAR*β*/*δ*, and PPAR*γ* subtypes with an EC_50_ value of 1.2, 1.7, and 0.08 *μ*M, respectively [33, 66, 67]. Research into chiglitazar's activity has significantly progressed over time. Initially considering chiglitazar as a PPAR*α*/*γ* dual agonist in 2006, Li and coworkers determined that in addition to improving insulin and glucose tolerance, chiglitazar's therapeutic effect on lipid homeostasis was discrete of the mechanism used by rosiglitazone and suggested that it was this distinction that would decrease multiple risk factors associated with selective PPAR*γ* full agonists [67]. Further research performed by He and coworkers in 2012 demonstrated chiglitazar's transactivating activity on each of the PPAR*α*, PPAR*β*/*δ*, and PPAR*γ* subtypes with a favorable distribution pattern, reclassifying chiglitazar as a PPAR pan-agonist. Through a comparison between chiglitazar and rosiglitazone in their work, He and coworkers studied chiglitazar's in vitro and in vivo activities highlighting the differential effects observed by the use of chiglitazar; a safer cardiac profile and no heart or body weight gain observed also provided evidence to support less risk for side effects [66]. Research by Pan and coworkers in 2017 further supported the benefits of chiglitazar's discrete mechanism through a comparison to two TZD drugs rosiglitazone and pioglitazone. In their study, Pan and coworkers described the interactions of chiglitazar as the full activation of PPAR*γ*, linked to insulin-related resistance gene expression, and a partial activation of PPAR*α* and PPAR*β*/*δ* that allows a balance between glucose and fatty acid uptake that positively affected other mechanisms implicated in insulin resistance and obesity [33]. Therefore, the current understanding is that chiglitazar's distinct interactions with the three PPAR subtypes will show enhanced efficacy and produce less long-term side effects than previously marketed T2DM drugs. Although chiglitazar is promising, molecular details on the full and partial activation mechanisms and the interactions and binding mechanism remain elusive. For example, Pan and coworkers molecular docking data showed that chiglitazar and the two TZD drugs bind differently to PPAR*γ* [33]. While rosiglitazone and pioglitzone form hydrogen bonds with PPAR*γ* Tyr473, chiglitazar forms hydrogen bonds with Ser289 and Glu343 instead. To verify the different binding modes, three point mutations (Tyr473Asp, Ser289Ala, and Glu343Ala) were studied. Unexpectedly, Tyr473Asp significantly diminished the transactivity of chiglitazar as well as rosiglitazone and pioglitzone [33]. Of the other two point mutations, only Ser289Ala attenuates the transactivity of chiglitazar which is different from rosiglitazone and pioglitzone. Clearly, molecular docking data does not completely explain the binding interaction between chiglitazar and PPAR*γ*.

By the use of a computational approach, research has studied protein-ligand interactions at the PPAR receptor that have successfully provided experimentally verified detailed structural information at the molecular level and identified a large number PPAR agonists [8, 22, 36, 38, 63, 68–80]. These include a number studies combining virtual screening with molecular modeling [64, 69], molecular docking [36], molecular dynamics (MD) simulations [81], and in vitro assays [38, 68] for further validation. Additionally, Ricci and coworkers have successfully used a dynamic network model coupled with principal component analysis to determine the allosteric pathways of the PPAR*γ*-RXR*α* nuclear receptor complex [78]. However, without an experimentally solved crystal structure of chiglitazar bound to the PPAR receptors, neither a detailed binding mode nor the structural and dynamic properties can yet be elucidated. To approach this in our study, we used molecular docking and molecular dynamics simulations to ascertain detailed structural and dynamic information at the molecular level to characterize the interactions of chiglitazar in complex with the PPAR*α*, PPAR*β*/*δ*, and PPAR*γ* receptors. Through visual inspection of structural clustering analysis, decomposition of the MM-GBSA binding energy by residue, and use of two-dimensional interaction diagrams, key residues involved in the binding of chiglitazar were identified and characterized for each complex system, supporting chiglitazar's activity as a pan-agonist and providing dynamic details to describe the underlying mechanism for fully activating PPAR*γ* and partially activating PPAR*α* and PPAR*β*.

## 2. Materials and Methods

### 2.1. Protein Preparation and Receptor Grid Generation

The crystal structures of the hPPAR*α* (PDB ID: 3VI8), hPPAR*β* (PDB ID: 3TKM), and hPPAR*γ* (PDB ID: 2PRG) receptor subtypes were obtained from the Protein Data Bank website. A sequence alignment of these receptors is presented in Figure 1. In Figure 1, the sequences were aligned using PPAR*γ* as a reference and each residue that differs is colored based on side-chain chemistry where red indicates residues D and E (acidic and hydrophilic); blue represents residues R, K, and H (basic and hydrophilic); green represents residues G, A, V, I, L, and M (neutral, hydrophobic, and aliphatic); orange represents residues F, Y, and W (neutral, hydrophobic, and aromatic); cyan represents residues S, T, N, and Q (neutral and hydrophilic); yellow represents residue C (primary thiol); and dark grey represents residue P (imino acid).

Using the Protein Preparation Wizard implemented in Maestro 10.2 [82], the following modifications were made to prepare the proteins: hydrogens and missing side chains were added, water molecules beyond 5 Å were deleted, and the proteins were optimized at pH 7.0. For hPPAR*γ*, only chain A was used. The optimized proteins underwent a restrained minimization to relax the protein structure using an OPLS3 force field [83]. To generate the receptor grid for each PPAR*α*, PPAR*β*/*δ*, and PPAR*γ*, the centroid of the crystal ligand was used as the active site [83]. Each receptor grid was generated using the default Van der Waals scaling factor of 1 and a partial charge cutoff of 0.25.

### 2.2. Ligand Preparation

The two-dimensional structures of all ligands (chiglitazar, rosiglitazone, pioglitazone, and WY-14643) were downloaded from the PubChem website. The three-dimensional ligand structures were prepared using Maestro Elements 2.2 implemented in the Maestro 10.2 software. The ionization/tautomeric states were generated at pH = 7 using EPIK which uses refined Hammett and Taft methodologies [83]. The lowest ionization/tautomeric state was selected. Ligand structure was relaxed via restrained minimization.

### 2.3. Ligand Docking

**Figure 3:**
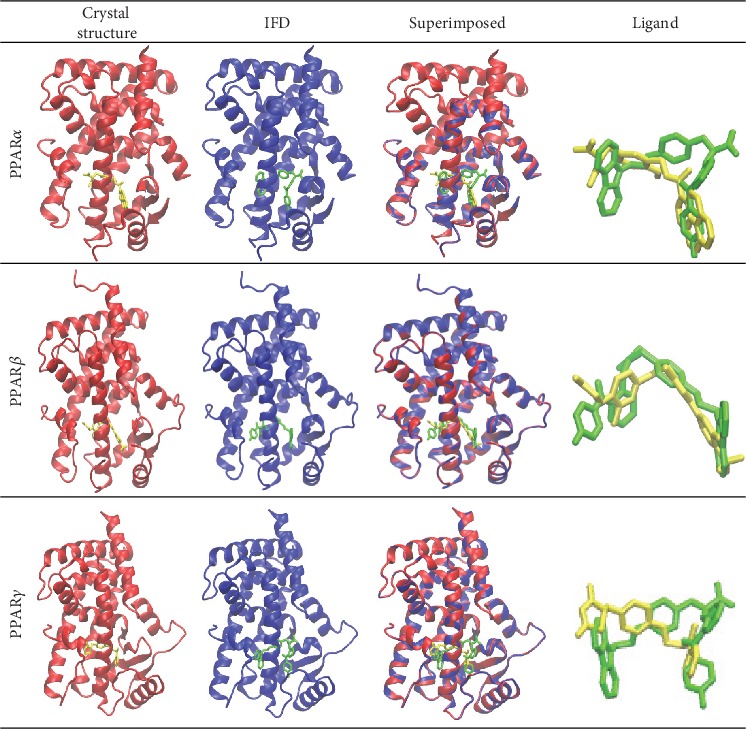
Structure comparison between the crystal complex and the induced fit docking of chiglitazar to PPAR*α* with a partial agonist APHM13 (PDB ID: 3VI8), PPAR*β* with a partial agonist GW0742 (PDB ID: 3TKM), and PPAR*γ* with a full agonist rosiglitazone (PDB ID: 2PRG). Chiglitazar is shown in green, whereas each crystal ligand is shown in yellow.

Glide XP docking provides a comprehensive and systematic search for the most favorable ligand-receptor conformations for a drug complex. Standard Glide dock was used to dock each crystal ligand into its respective receptor grid (3VI8 (hPPAR*α*), 3TKM (hPPAR*β*), and 2PRG (hPPAR*γ*)) using Glide XP scoring functions under default parameters [84, 85]. Following the same protocol, prepared chiglitazar was docked into each receptor, then additional induced fit docking protocol was used to optimize the docking pose. The results from the induced fit docking of chiglitazar and the initial crystal structure are shown in Figure 3.

### 2.4. Molecular Dynamics Simulation Setup and Production Runs

Using the prepared receptor-ligand complexes, six molecular dynamics simulation systems were created: the crystal ligands in complex with PPAR*α*, PPAR*β*, and PPAR*γ* and chiglitazar in complex with PPAR*α*, PPAR*β*, and PPAR*γ*. Each system was solvated in an orthorhombic water box using the SPC water model with a 10 Å water buffer [86]. To neutralize the systems, Na^+^ ions were added with a salt concentration of 0.15 M NaCl. After successful solvation of each system, the OPLS3 force field [83] was used to represent the receptor-ligand complex.

For each system, the default relaxation protocols were followed in the Desmond simulation package [87]. Detailed relaxation procedures follow our early work [88–90]. After the relaxation step, three independent 1000 ns production runs were carried out for each system, leading to a total of 3000 ns for each system.

### 2.5. Convergence of Simulation

**Figure 4:**
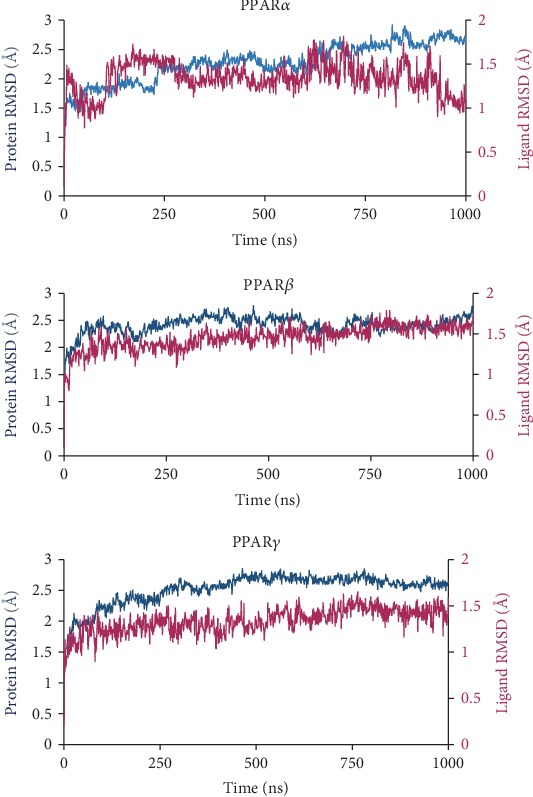
Average root mean square deviation (RMSD) plot for the three MD simulation runs of each protein-ligand complex over the length of the trajectory. The C*α*-RMSD for the protein (shown in blue) and ligand RMSD (shown in red) is based on the initial protein alignment.

In order to check the convergence of the simulation systems and determine whether the complex systems had reached a steady state, the C*α* (protein) and ligand RMSD was generated using the average of all three simulation runs for the systems (Figure 4 and [Supplementary-material supplementary-material-1]). From the RMSD plot, we see that the simulation systems reach a steady state around 500 ns; thus, the last 500 ns were used for subsequent analysis.

### 2.6. Simulation Interaction Diagram (SID) Analysis

**Figure 5:**
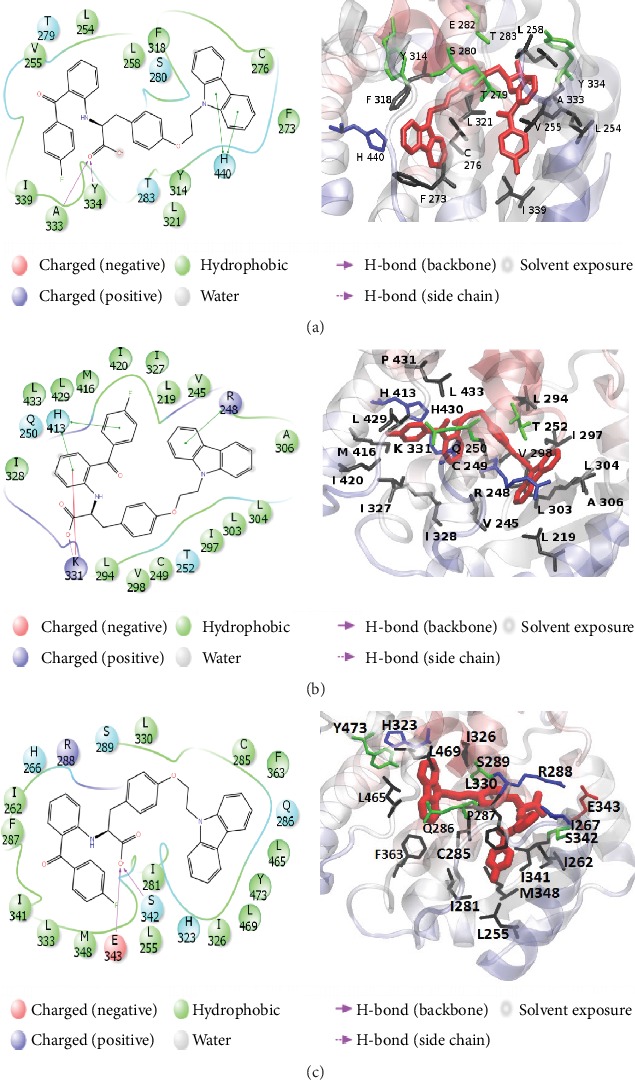
Two- and three-dimensional interaction diagrams of chiglitazar's binding pose in complex with the PPAR*α* (a), PPAR*β* (b), and PPAR*γ* (c) receptors from the most abundant cluster of the combined MD simulation.

**Figure 6:**
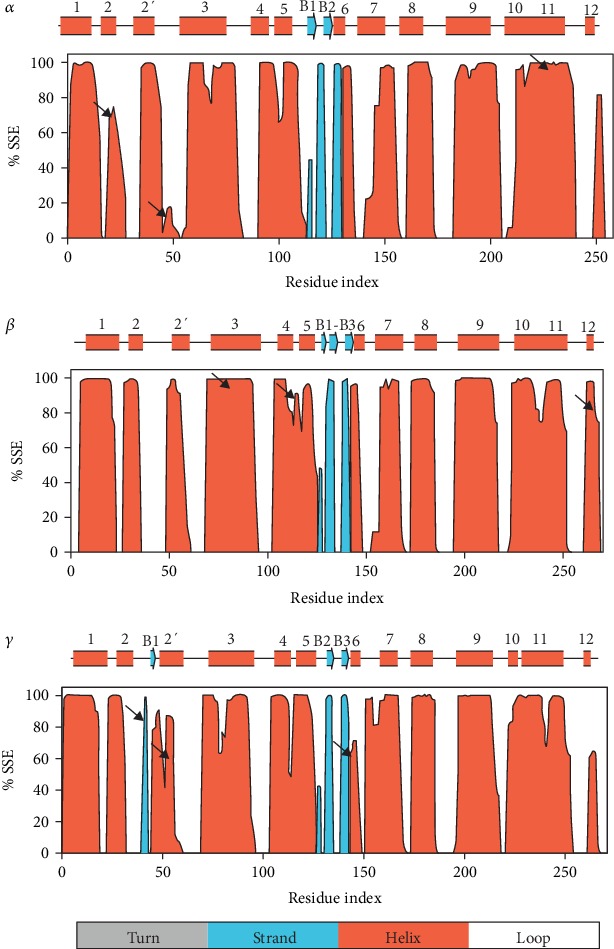
Illustrations of residue index as well as the %SSE in hPPAR receptors: PPAR*α* (3VI8), PPAR*β* (3TKM), and PPAR*γ* (2PRG). Protein receptor secondary structure elements and their composition percentages are annotated.

**Figure 7:**
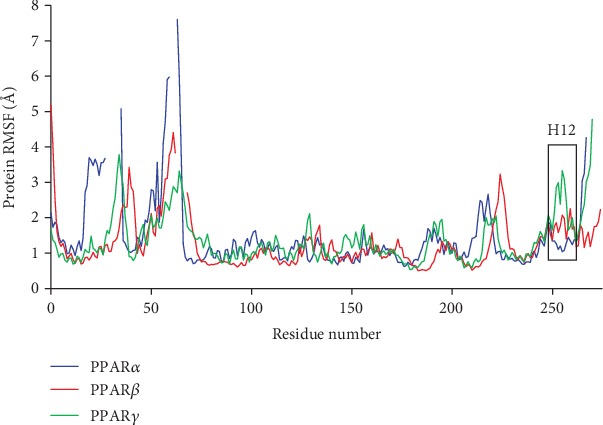
Average receptor protein C*α* RMSF for all trajectories of PPAR*α* (red), PPAR*β* (blue), and PPAR*γ* (green), presented with the sequences aligned.

**Figure 8:**
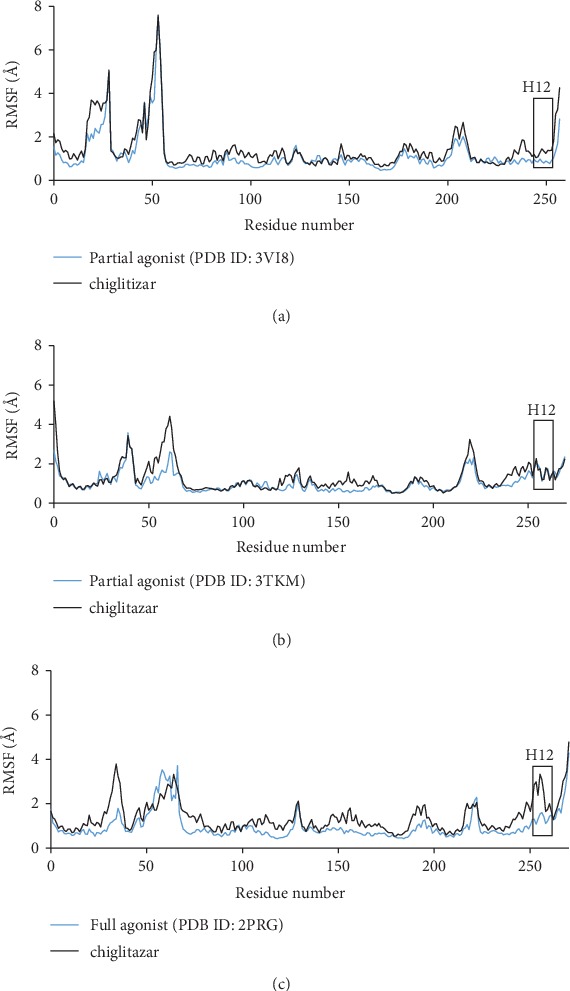
Root mean square fluctuations of chiglitazar from our MD simulation compared to the crystal ligand systems for PPAR*α* (a) (PDB ID: 3VI8), PPAR*β* (b) (PDB ID: 3TKM), and PPAR*γ* (c) (PDB ID: 2PRG) where the date for the crystal ligands are in blue and chiglitazar's data is in black.

**Figure 9:**
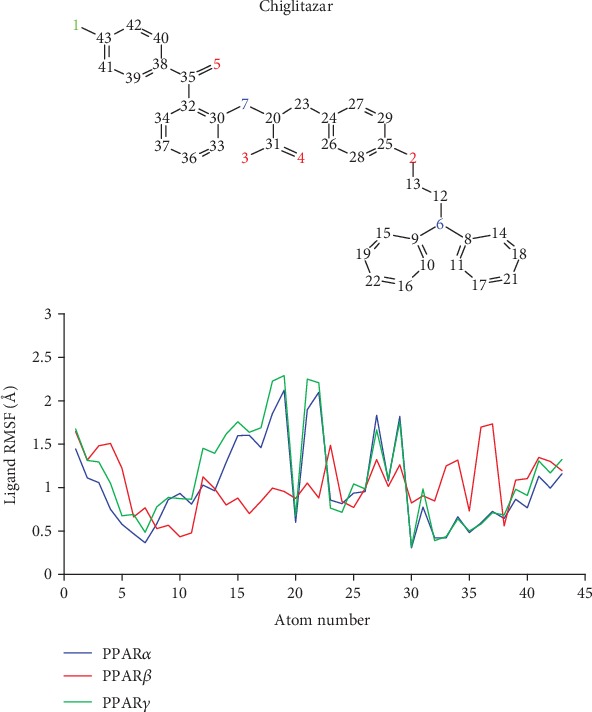
Ligand RMSF diagrams of chiglitazar in complex with PPAR*α* (red), PPAR*β* (blue), and PPAR*γ* (green) in the combined trajectories.

The SID tool within Desmond was used to analyze the interactions between the protein and ligand in each of the simulation systems. We also included 2D interaction diagrams (Figure 5), secondary structure changes (Figure 6), protein and ligand root mean square fluctuation (RMSF) (Figures 7–9), protein-ligand contacts ([Supplementary-material supplementary-material-1]), and torsional angle profiles ([Supplementary-material supplementary-material-1]). The protein and ligand RMSF, 2D interaction diagrams, secondary structure diagram, torsional angle plots, and 2D interaction profiles were generated using a combined trajectory of the simulation runs.

### 2.7. Clustering Analysis

**Figure 10:**
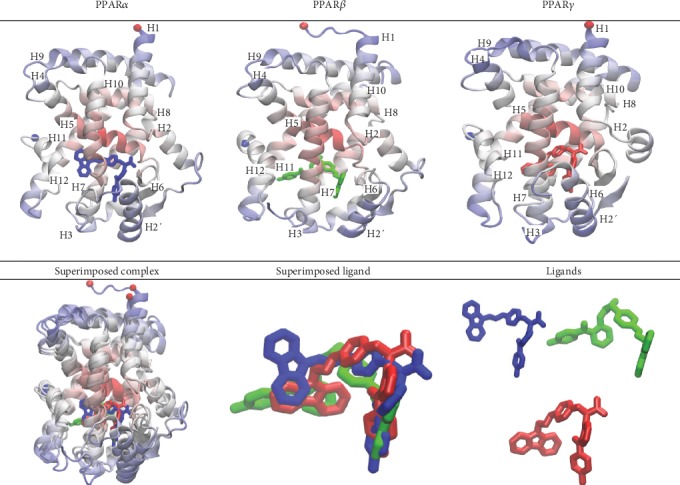
Structural comparison of the most abundant complex structure of each PPAR*α* (3VI8), PPAR*β* (3TKM), and PPAR*γ* (2PRG) including the superimposed complex. Chiglitazar is represented in blue, green, and red for the PPAR*α*, PPAR*β*, and PPAR*γ* complexes, respectively, and each receptor is colored based on mobility where blue is the most mobile and red is the least.

The trajectory clustering tool implemented in Desmond [91] was used to group together the complex structures of the simulation period for each system based on structural similarity. The merging distance cutoff was set to 2.5 Å for the hierarchical clustering with the average linkage method [91]. The structure with the largest number of neighbors in the structural family (centroid structure) was used to represent the structural family. These centroid structures (>1% of the total structure population) are presented in Figure 10. For further analysis, we repeated our clustering analysis technique on our combined trajectory for each system ([Supplementary-material supplementary-material-1]).

### 2.8. Binding Energy Calculations

Molecular Mechanics-General Born Surface Area (MM-GBSA) binding energies were calculated for the last 200 nanoseconds of the combined trajectory for each system (Table 1). For this calculation, the OPLS3 force field, VSGB 2.0 solvation model, and the default Prime protocol were used to separately minimize the receptor, ligand, and receptor-ligand complex using the equation for the total binding free energy: Δ*G*_(bind)_ = *E*_complex (minimized)_–(*E*_ligand (minimized)_ + *E*_receptor (minimized)_). The components (Coulombic+H-bond+GB solvation+van der Waals+*π*-*π* packing+self-contact+lipophilic) were further merged into the following three groups to provide deeper insight into the binding process: *E*_electrostatic_, *E*_vdW_, and *E*_lipophilic_ (where *E*_electrostatic_ = *E*_coulombic_ + *E*_H−bond_ + *E*_GB−solvation_ and *E*_vdW_ = *E*_vdW_ + *E*_pi−pi stacking_ + *E*_self−contact_).

### 2.9. Normal Mode Analysis

The combined trajectories were used in the VMD Normal Mode Wizard [92] to generate principal component analysis (PCA) of the top 5 modes, and their associated root mean square fluctuation graphs were generated ([Supplementary-material supplementary-material-1]). The antistrophic network models were generated using the ANM 2.1 webserver [93].

### 2.10. Dynamical Network Model

**Figure 11:**
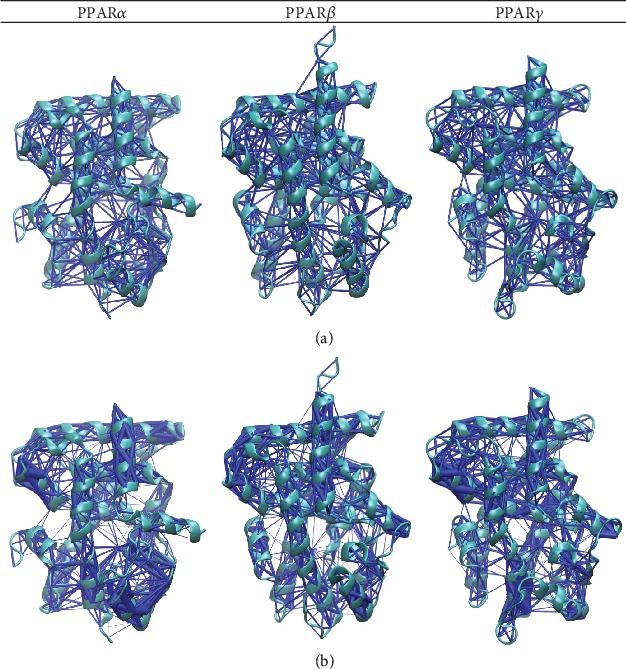
Unweighted (a) and weighted (b) network models of PPAR*α*, PPAR*β*, and PPAR*γ*.

The combined trajectories of each system were used to generate a dynamic network model, defined as a set of nodes connected by edges, [94–98] using the NetworkView plugin [94] in VMD [99]. For each system, we generated a contact map (Figure 11(a)) which added an edge between nodes whose heavy atoms interacted within a cutoff of 4.5 Å for at least 75% of the MD simulation time. The early study has shown that the effect of the cutoff parameter on the network properties is minor when the cutoff distance~4.5 Å [100]. In this contact map, the edge distance was derived from pairwise correlations [94] using the program Carma [101], which defines the probability of information transfer across a given edge using the following equation:

(1) $$\begin{array}{c} C_{ij}=\frac{\langle \left(\Delta \vec{r_{l}}\left(t\right)\cdot \Delta \vec{r_{j}}\left(t\right)\right)\rangle }{{\left(\langle \left(\Delta \vec{r_{l}}{\left(t\right)}^{2}\right)\rangle \langle \Delta \vec{r_{j}}{\left(t\right)}^{2}\rangle \right)}^{1/2}}. \end{array}$$

In the pairwise correlation equation (*C*_*ij*_), the term $\overset{⃑}{\;r_{i}}\left(t\right)$ is the positon of the atom used to define the node “*i*” and $∆\overset{⃑}{r_{i}}\left(t\right)=\overset{⃑}{r_{i}}\left(t\right)-\langle \overset{⃑}{r_{i}}\left(t\right)\rangle$ which represents the change in the position of this atom at two different times. Using the pairwise correlation data in the dynamic network model, the edges are weighted (*w*_*ij*_) between two nodes *i* and *j* using the following calculation: *w*_*ij*_ = −log(|*C*_*ij*_|). The weight of the edge represents the probability for information to transfer across the edge between the two nodes; thus, a thicker edge represents a higher probability of information transfer.

**Figure 12:**
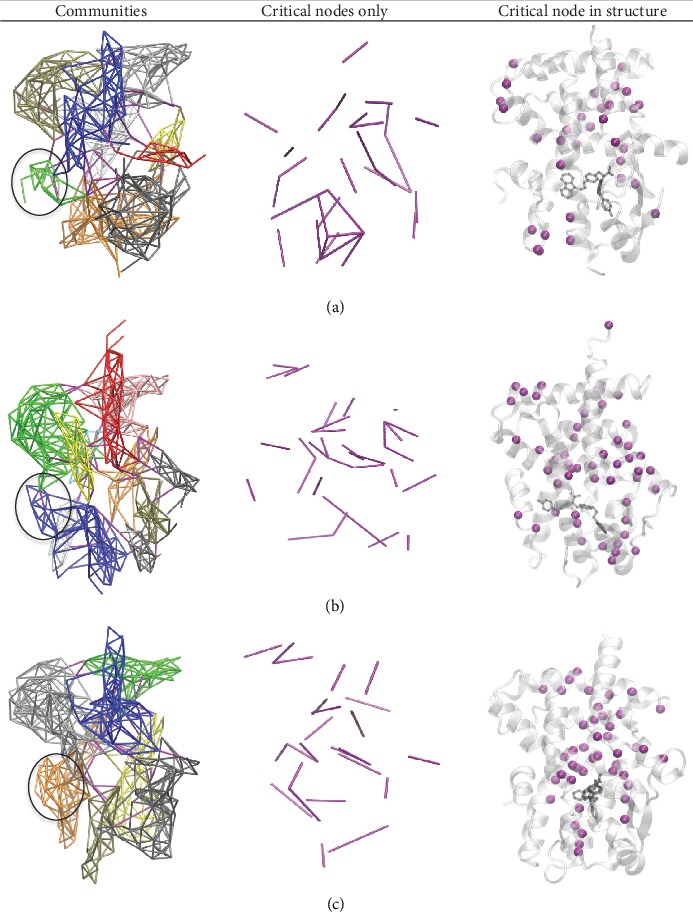
Model of structural communities separated by color with critical nodes shown in purple for PPAR*α* (a), PPAR*β* (b), and PPAR*γ* (c). Helix 12 is circled.

Each network was then further grouped into subnetworks, termed communities, based on groups of nodes with stronger and more frequent connections to each other. This was done by applying the Girvan-Newman algorithm to the original network [102]. Critical nodes that connect communities to another were also identified (Figure 12).

## 3. Results

### 3.1. Docking Revealed Subtle Differences in the Binding Poses of Chiglitazar Compared to the Crystal Structures

For the PPARs, several structural features are conserved amongst the receptor subtypes (PPAR*α*, PPAR*β*, and PPAR*γ*) which include the activation function 1 (AF-1), DNA binding domain (DBD), activation function 2 (AF-2), and ligand-binding domain (LBD) [58, 103, 104] whose sequence alignment is presented in Figure 1 and shows a 65% homology amongst the three subtypes [15, 27, 104]. Based on the current experimental understanding of chiglitazar's activity on each receptor subtype's LBD, the following receptors were used in our study ([Supplementary-material supplementary-material-1]): a partial agonist (APHM13) system of PPAR*α* (PDB ID: 3VI8) [105], a partial agonist (GW0742) system of PPAR*β* (PDB ID: 3TKM) [106], and a full agonist (rosiglitazone) system of PPAR*γ* (PDB ID: 2PRG) [107]. To validate the docking protocol, the crystal ligands were successfully docked back to their respective receptors, overlapping well with the original crystal pose (data is not shown). Using identical Glide XP docking protocol followed by the induced fit docking protocol, we docked chiglitazar into each of the PPAR*α*, PPAR*β*, and PPAR*γ* receptors and compared the binding of chiglitazar to the crystal ligands (Figure 3). Though subtle differences in binding poses were observed between chiglitazar and the crystal ligands, the overall agreement provided additional validation for the docking procedure used in this study.

### 3.2. MD Simulation

We used a combined trajectory from three independent simulation trajectories for our MD analysis. The protein-ligand RMSD plot (Figure 4) of the combined trajectories shows that both the protein and ligand remained stable throughout the simulation runs. The C*α* of PPAR*α* experiences a gradual increase in deviation for the entirety of the simulation period, whereas chiglitazar, in complex with PPAR*α*, undergoes more prominent deviations until roughly 300 ns averaging at ~1.25 Å for the last 750 ns. For PPAR*β*, C*α* experiences gradually increasing deviations until ~600 ns, where it maintained a deviation of ~2 Å for the remainder of the simulation period; as for chiglitazar in complex with PPAR*β*, only minor deviations were observed throughout the length of the trajectory maintained at ~1.5 Å. PPAR*γ*'s C*α* showed a gradual increase in deviation until roughly 500 ns, where it maintained a deviation of ~2.6 Å; chiglitazar in complex with PPAR*γ* showed very little deviation across the simulation period maintaining a deviation of roughly 1.4 Å for the entirety of the simulation period. In addition to this, the RMSD of the simulated crystal systems for PPAR*α*, PPAR*β*, and PPAR*γ* are presented in [Supplementary-material supplementary-material-1], and the crystal complex structures are well maintained in the MD simulation. The binding pose of chiglitazar from the induced fit binding and the MD simulation are presented in supporting [Supplementary-material supplementary-material-1] and shows very minor changes in the position of chiglitazar for each system.

### 3.3. MM-GBSA Binding Energy Calculations Predicted PPAR*γ* Was Most Energetically Favorable Followed by PPAR*α* and PPAR*β*

The MM-GBSA binding energy calculations (Table 1) showed that chiglitazar binds most favorably to PPAR*γ* (-144.6 kcal/mol) followed by a comparable binding interaction with PPAR*α* (-138.0 kcal/mol) and PPAR*β* (-135.9 kcal/mol), where PPAR*γ* binds more strongly to chiglitazar than to PPAR*α* by 6.6 kcal/mol and to PPAR*β* by 8.7 kcal/mol. Van der Waals interactions contributed the most to the binding of PPAR*γ* (-87.9 kcal/mol), PPAR*α* (-82.9 kcal/mol), and PPAR*β* (-76.4 kcal/mol). However, the lipophilic term also contributed greatly to the binding of chiglitazar to PPAR*γ* (-71.3 kcal/mol), PPAR*α* (-67.5 kcal/mol), and PPAR*β* (-71.9 kcal/mol).

### 3.4. The Clustering Analysis Identified the Major Binding Poses of Each Complex System

As described in Materials and Methods, the major binding pose from each complex system was identified using structural clustering of the combined trajectories [91], where the most abundant structure was used to represent the structural family (Figure 10). Clustering of the combined trajectory ([Supplementary-material supplementary-material-1]) revealed three major clusters for PPAR*α* (48.9%, 31.9%, and 18.1%), two clusters for PPAR*β* (98.8% and 1.09%), and one cluster for PPAR*γ* (100%). Superimposition and inspection of the receptor complexes show that although there is a good overlap of receptors themselves, the position of chiglitazar in complex with each receptor reveals subtle differences that may be responsible for the differences in binding energies between systems. Chiglitazar in complex with both PPAR*γ* and PPAR*α* was positioned with the carbazole side chain wrapped around the left side (respective for the point of view used in this study) of helix 3, whereas in the PPAR*β* system, chiglitazar positioned the 4-flourobenzophenone side chain around the left side of helix 3. The carbazole side chain of chiglitazar shows enhanced interactions with helices 3, 7, and 11 in the PPAR*γ* and PPAR*α* systems, whereas the PPAR*β* conformation allows the least potential for interaction on the lower right region of the binding pocket (H6, H7, and H2′).

### 3.5. The Two-Dimensional Protein-Ligand Interaction Diagrams Revealed Key Residues Involved in the Binding of Chiglitazar to PPAR*α*, PPAR*β*, and PPAR*γ*

Key residues that maintained interactions with chiglitazar within 2 Å for at least 30% of the simulation period were identified to be involved in the binding of chiglitazar to each receptor subtype using the Desmond Simulation Interaction Diagram (Figure 5).

For PPAR*α*, the major interactions included hydrophobic interactions between Tyr334 and the oxygen at position 4 of chiglitazar for 97% of the simulation period, as well as the aromatic ring starting at position 30 for 56% of the simulation period (see Figure 9 for reference to numbering). Through an interaction with water, Gly335 maintained interaction with chiglitazar for 39% of the simulation period and Lys358 for 38% of the simulation period. Hydrogen bonding between the oxygen at position five and the hydrogen attached to the nitrogen on position 7 also occurred for 67% of the simulation period. Hydrophobic interactions also played a key role in the binding of chiglitazar, where residues Cys275, Cys276, Tyr314, Leu321, and Val332 all interacted with chiglitazar for at least 30% of the simulation period.

For PPAR*β*, the key interacting residue was Lys331, which interacted with the oxygen at positions 3 and 4 for 34% and 50% of the simulation run, respectively, while also interacting with the oxygen at position 3, through water, for 39% of the simulation period. His413 interacted with the oxygen at position 4 for 34% of the simulation period as well as maintained hydrophobic interactions with the aromatic ring at position 30. Hydrogen bonding between the oxygen at position five and the hydrogen attached to the nitrogen on position 7 also occurred for 30% of the simulation period. In addition, Cys249 and Val305 maintained hydrophobic interactions with the carbazole side chain for at least 30% of the simulation period.

For PPAR*γ*, Glu343 interacted with the oxygen at positon 4 for 83% of the simulation period, and through an interaction with water Lys265 also interacted with the oxygen at position four for 30% of the simulation period. Lys367 interacted directly with the pyrrole core of the carbazole side chain for 42% and one of the aromatic rings for 46% of the simulation period. Hydrogen bonding between the oxygen at position five and the hydrogen attached to the nitrogen on position 7 also occurred for 30% of the simulation period. Additionally, Ile431, Leu330, and Phe282 maintained hydrophobic interactions with chiglitazar for at least 30% of the simulation period. In addition, [Supplementary-material supplementary-material-1] provides a histogram plot summarizing the type and fraction of interaction of each major residue in the combined trajectory systems.

### 3.6. Secondary Structure Analysis Reveals Differences in Helices 2, 2′, 3, 5, and 11 between Systems

The secondary structure analysis (Figure 6) represents the residue index and the percentage of the secondary structure element abundance for the combined trajectory analysis. Changes in the SSE are represented by dips and reflect bends in the transmembrane regions. Black arrows are used to represent major differences in the secondary structure between systems. The PPAR*α* complex differs from both PPAR*β* and PPAR*γ* in helices 2, 2′, and 11. The PPAR*β* complex differs from both PPAR*α* and PPAR*γ* in helices 3, 5, and 12, and the PPAR*γ* complex differs from both PPAR*α* and PPAR*β* in helices 2′ and 6.

### 3.7. The Protein C*α* Root Mean Square Fluctuation Confirms the Overall Stability of PPAR*α*, PPAR*β*, and PPAR*γ*

Overall, the protein RMSF (Figure 7) for each system were comparable. The RMSF for each system remained relatively low for the residues of the core. The most significant differences were within the first 60 residues of each receptor with PPAR*α* showing slightly larger fluctuations around residues 20 to 40 and from 50 to 60. Small fluctuations were present in the last 60 residues of each system, which may correspond to the movement of the terminal helix 12. However, differences were also observed for helix 11 between systems. The RMSF broken down by helix is presented in a supporting document ([Supplementary-material supplementary-material-1]).

### 3.8. The Protein Root Mean Square Fluctuation of Chiglitazar in Complex with Each Receptor Subtype as Comparable to the Crystal Systems for Each PPAR*α*, PPAR*β*, and PPAR*γ*

In order to better understand the relative fluctuation of chiglitazar, as compared to known full and partial agonists, we compared the RMSF of our combined MD simulation runs to a simulation run of the crystal ligand system for each PPAR*α*, PPAR*β*, and PPAR*γ* (Figure 8). For PPAR*α* with a partial agonist (APHM13), chiglitazar shows comparable fluctuations when compared to the crystal system. The RMSF of chiglitazar and the crystal system for PPAR*β* with a partial agonist (GW0742) are very comparable with chiglitazar showing a slightly higher fluctuation in the 2′ helix as compared to the crystal system. For PPAR*γ*, there are slightly higher fluctuations overall, as compared to the crystal system with a full agonist (rosiglitazone). Specifically, out of the three subtypes, PPAR*γ* shows the highest fluctuation of helix 12 when compared to the crystal systems.

### 3.9. The Ligand Root Mean Square Fluctuation for Chiglitazar in Complex with PPAR*α*, PPAR*β*, and PPAR*γ* Shows Minor Fluctuations

With the largest fluctuation (Figure 9) of chiglitazar being ~2.25 Å in the case of PPAR*γ* around position 18, the overall fluctuation of chiglitazar remained minimal across the combined trajectories. The overall ligand RMSF of chiglitazar was very comparable for the PPAR*α* and PPAR*γ* systems, where PPAR*γ* experienced ~0.25 Å greater fluctuations on average. The PPAR*β* complex showed the lowest ligand RMSF averaging ~1 Å. Additionally, a lower exposure to the solvent of chiglitazar in complex with PPAR*β* might explain the lower ligand RMSF observed when compared to PPAR*α* and PPAR*γ*.

### 3.10. Torsional Angle Distribution Profile of the Ligand Reveals Key Differences in the Major Binding Pose of Chiglitazar in Complex with PPAR*α*, PPAR*β*, and PPAR*γ*

The torsional angle distribution profile of the ligand ([Supplementary-material supplementary-material-1]) presents differences of PPAR*β* when compared to PPAR*α* and PPAR*γ* consistent with PPAR*β* having a major binding pose that was fundamentally different from the comparable poses of PPAR*α* and PPAR*γ*. Most notably, PPAR*β* differed from PPAR*α* and PPAR*γ* in the two bonds nearest the carbazole side chain (depicted in purple and brown), as well as for both the angle connecting the carboxylic acid at positon 31 (dark green) and for the angle connecting the aromatic ring at positon 32 (light green).

### 3.11. The Dynamic Network Model Reveals Key Features in the Overall Connectivity of PPAR*α*, PPAR*β*, and PPAR*γ*

For each PPAR*α*, PPAR*β*, and PPAR*γ*, the backbone C*α* residues were used to generate an unweighted network model (Figure 11(a)) where edges connect two residues that came in contact within 4.5 Å for over 70% of the length of the simulation period. The edges were then quantified using a correlation matrix so that two residues with high correlation would have smaller edges and those with low correlation would have larger edges, as represented in the weighted network model (Figure 11(b)). For each PPAR*α*, PPAR*β*, and PPAR*γ*, the unweighted networks had very similar connectivity, as expected for their similar structures and sequence. There were subtle differences when looking at the weighted network; for example, in the PPAR*α* system, the area with the lowest correlation was the omega loop; for PPAR*γ*, these areas were in helices 3 and 4/5; while for PPAR*β*, the weighted network showed roughly equal edges throughout so no notable correlations were observed.

Using the weighted network model, communities were generated which grouped together residues that interacted more frequently and stronger than the residues in other communities (Figure 12). Critical residues were also identified as residues that were most essential in the collective motions of different communities (Figure 12 and [Supplementary-material supplementary-material-1]). Most notably from the network analysis were the differences in communities around helix 12 where PPAR*α* (residues 548-462), PPAR*β* (residues 431-438), and PPAR*γ* (residues 467-473) had helix 12 involved in completely different communities. Specifically, for PPAR*α*, helix 12 is completely separate from other communities but shows some critical edges linking its communication network to the lower portion of helix 3. For PPAR*β*, helix 12 forms a large community with the bottom of helix 3, but there are also minor connections to helix 4. Then for PPAR*γ*, the major community formed with helix 12 includes helix 11 in its entirety. Although very comparable, for the critical nodes, it appears that those of PPAR*γ* were slightly more focused around the binding pocket as compared to the greatly spread out nodes of PPAR*α* and PPAR*β*.

### 3.12. A Principal Component Analysis of PPAR*α*, PPAR*β*, and PPAR*γ* Revealed Significant Differences in Helix 12

**Figure 13:**
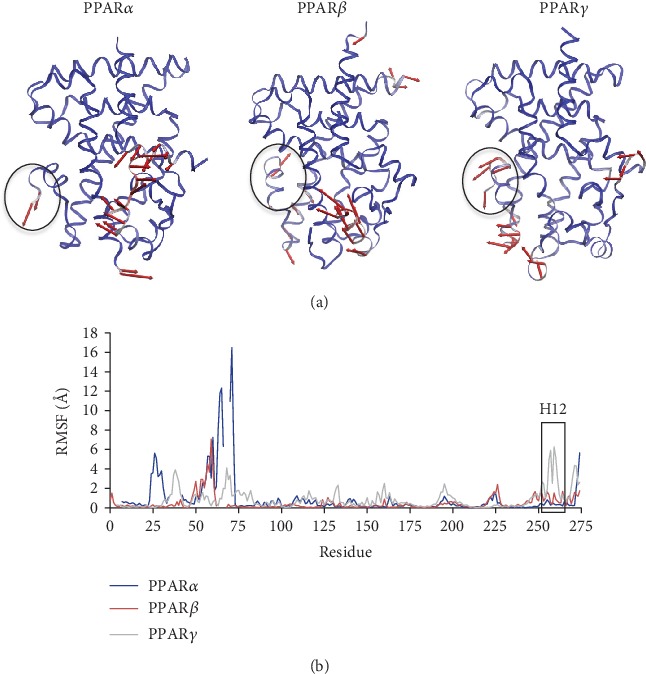
Snapshots of Mode 1 from the trajectory-based principal component analysis (a) and root mean square fluctuation of Mode 1 (b). For the principal component analysis, vectors (red) that are longer than 3.5 Å are shown.

A principal component analysis of the combined trajectories calculated the lowest energetic modes of the global motions of each PPAR*α*, PPAR*β*, and PPAR*γ* ([Supplementary-material supplementary-material-1]). The lowest vibrational mode, Mode 1 (Figure 13(a)), showed clear differences at helix 12 where both PPAR*α* and PPAR*γ* are moving outward away from the receptor, whereas PPAR*β* is moving upward toward the receptor. Consistent with the principal component analysis, the RMSF (Figure 13(b)) of this mode showed that at helix 12, PPAR*γ* had the highest fluctuations, followed by PPAR*β* and then PPAR*α*. The top five modes of the combined trajectories, which include two additional PCA analyses from the last 500 ns of our combined trajectories, split into two 250 ns blocks ([Supplementary-material supplementary-material-1]), and the RMSF plots of the top five modes of the combined trajectories ([Supplementary-material supplementary-material-1]) are presented in a supporting document.

## 4. Discussion

With diabetes affecting over 420 million people worldwide, there is a dire need for safe and effective treatment. The current medications available, thiazolidinediones (TZDs), are PPAR*γ* full agonists associated with dangerous side effects including hepatotoxicity, increased risk for cardiovascular failure, myocardial infarction, increased risk for bladder cancer, and body weight gain. It has been shown that the overactivation of PPAR*γ* is likely the major causative factor for the negative side effects [64]. Despite this, the development of new PPAR agonists are still of great interest because of the unique and promising features of this class of drug, like the ability to directly target insulin resistance and provide a more durable glycemic (HbA1c) control when compared to other antidiabetic medications [33]. With this in mind, the use of nonselective PPAR pan-agonists that interact with PPAR*α*, PPAR*β*, and PPAR*γ* with a balanced activation profile is a promising new strategy for the treatment of T2DM.

Chiglitazar is a pan-agonist to the PPAR receptors which has shown promising results in both *in vitro* and *in vivo* experiments for the treatment of Type 2 Diabetes Mellitus (T2DM). Currently in stage III clinical trials, chiglitazar does not produce harmful and potentially fatal side effects, like cardiac toxicity, that other PPAR selective medications have produced. However, since there is no crystal structure of chiglitazar in complex with any subtype of the PPAR receptor, the detailed structural and dynamic information needed to fully understand the mechanism involved remains elusive. Understanding and further exploiting the mechanism of chiglitazar toward the PPAR receptors offer a unique opportunity to further expand this new generation of T2DM medications. To this end, we modeled the binding of chiglitazar in complex with PPAR*α*, PPAR*β*, and PPAR*γ* using molecular docking; performed molecular dynamics simulations to analyze the specific binding interactions of each system including the major helices and residues involved; presented the major binding pose as extracted from our multiple trajectory clustering analysis; quantified the binding interactions with our MM-GBSA binding energy analysis; provided dynamic insight into the complexes using a network model; and characterized the global motions of the receptor complexes using PCA.

Experiments have shown that chiglitazar acts on the PPAR*α*, PPAR*β*/*δ*, and PPAR*γ* subtypes with an EC_50_ value of 1.2, 1.7, and 0.08 *μ*M, respectively [33, 66, 67]. From our MM-GBSA binding energy analysis, we determined that the relative order of binding favorability was to PPAR*γ* (-144.6 kcal/mol), followed by PPAR*α* (-138.0 kcal/mol) and PPAR*β* (-135.9 kcal/mol). Our relative order of stability matched the EC_50_ values reported in experiments and validated the accuracy of our calculations. Additionally, when compared to our MM-GBSA binding energy calculations of PPAR*α*, PPAR*β*/*δ*, and PPAR*γ* in complex with their crystal ligands ([Supplementary-material supplementary-material-1]), chiglitazar bound more favorably by -12.2, -12.6, and -43.6 kcal/mol than the PPAR*α*, PPAR*β*/*δ*, and PPAR*γ* crystal complexes, respectively ([Supplementary-material supplementary-material-1]). Clustering of the combined trajectory ([Supplementary-material supplementary-material-1]) revealed three major clusters for PPAR*α* (48.9%, 31.9%, and 18.1%), two clusters for PPAR*β* (98.8% and 1.09%), and one cluster for PPAR*γ* (100%). From this, we observe that the binding pose of chiglitazar was fundamentally different in the PPAR*β* system, for both the docking and MD simulation, when compared to PPAR*α* and PPAR*γ*. Specifically, for the PPAR*β* system, we observed the 4-flourobenzophenone side chain of chiglitazar wrapped around the left of helix 3 (respective for the point of view used in this study), rather than the carbazole side chain as observed in both PPAR*α* and PPAR*γ*.

Pan and coworkers of Shenzhen Chipscreen Biosciences Ltd. performed a molecular docking of rosiglitazone, pioglitazone, and chiglitazar in complex with PPAR*γ* based on crystal structure (PDB ID: 2PRG, 2XKW) [33]. Pan and coworkers' docking results showed that chiglitazar and TZD-class compounds differentially bind to PPAR*γ* based on the fact that chiglitazar did not show hydrogen binding to Tyr473 or His323, key interactions of PPAR*γ* full agonists. Instead, Pan and coworkers' docking identified that chiglitazar's major interactions were with Ser289, Arg288, and Glu343. Using a transactivity assay, Pan and coworkers further examined the different binding poses using serial site-directed mutations of Tyr473, Ser289, and Glu343 replacing these residues with Asp, Ala, and Ala, respectively. It is unexpected that the transactivity of chiglitazar was comparable to rosiglitazone and pioglitazone, with the most notable difference being the Ser289 mutation. Despite the docking of chiglitazar to PPAR*γ* not showing a hydrogen bond interaction with Tyr473, the transactivity was diminished upon mutation of Tyr473, showing experimental evidence of full agonist activity, a result that Pan and coworkers were not expecting based on the docking pose [33]. We examined our docking results of chiglitazar into the PPAR*γ* receptor ([Supplementary-material supplementary-material-1]), which is very similar to the ones obtained by Pan and coworkers. It also showed the major hydrogen bond interactions to be with Arg288 and Glu343. However, when compared to the 2D interaction diagrams generated based on our MD simulation results, chiglitazar interacted with each residue Arg288, Tyr473, Ser289, and Glu343, amongst others (Figure 5 and [Supplementary-material supplementary-material-1]). Thus, we believe that the lack of interaction between chiglitazar and Tyr473 could be a flaw due to the lack of dynamics in the molecular docking method, and a more advanced MD simulation was able to obtain a more complete interaction between the drug and the protein. Assuming this, the MD-derived binding pose and major interactions identified in our study help to explain Pan and coworkers' unexpected full agonist transactivation pattern and further support chiglitazar's full agonist activity.

As compared to the crystal structures of known full and partial agonists of PPAR*α*, PPAR*β*, and PPAR*γ* (Figure 8), the RMSF of chiglitazar from our combined MD simulation runs showed comparable fluctuations, with some regions showing slightly higher fluctuations than others. For PPAR*α*, there were slightly higher fluctuations in the beta region, where the omega loop, helix 2, and 2′ helix fluctuate slightly more than the crystal structure. This larger fluctuation could be due to the less conjugated side chain of chiglitazar in this pocket, which may undergo slightly weaker van der Waals stabilization, as compared to the crystal ligand. In addition, the smaller fluctuations of the crystal system may be because chiglitazar's binding pose is positioned slightly closer to helix 12, whereas the crystal ligand is slightly closer to the beta region and is able to form slightly stronger interactions in this region. Despite the differences in fluctuations observed in the omega region, the comparison to the crystal system supports chiglitazar's role as a partial agonist to PPAR*α*. The RMSF of chiglitazar and the crystal system for PPAR*β* are very comparable, with chiglitazar showing a slightly higher fluctuation in the 2′ helix as compared to the crystal system, also supporting chiglitazar's role as a partial agonist at PPAR*β*. For PPAR*γ*, there are slightly higher fluctuations overall, as compared to the crystal system. Specifically, out of the three subtypes, PPAR*γ* shows the highest fluctuation of helix 12 when compared to the crystal systems. Generally, a weaker binding ligand will show higher fluctuations, but there have been exceptions to this, as exemplified by Dhankik et al. [108]. Despite the higher fluctuations of chiglitazar at PPAR*γ* in our study, it was a much stronger binder to PPAR*γ* as compared to the crystal ligand which presents another example of this exception. Overall, the comparison of PPAR*γ* to the crystal system does support its activity as a full agonist at PPAR*γ*, where the slightly higher fluctuations may be due to the differences in binding poses when compared to the crystal system.

Decomposition of MM-GBSA by residue, based on structure alignment.

With the relative order of stability validated with experimental findings, we set out to gain insight into the interactions that are involved in chiglitazar's full activation of PPAR*γ* but partial activation of PPAR*α* and PPAR*β*. It is known that all three hPPAR subtypes have a large Y-shaped pocket including three subarms (arms I, II, and III) [106] with approximately 1300-1440 Å^3^ volume to accommodate the ligand [58]. Of the three arms, studies have identified that full agonists of PPAR*γ* primarily occupy arm I (helices 3, 5, 11, and 12), with key interactions with residues H323, H449, and Y473 [109, 110] but also including Cys285 [64], Ser289, and Tyr327 [30, 64, 69], whereas partial agonists maintain primary interactions in arm II (helices 2′, 3, 6, and 7) and arm III (2, 3, 5, *β*-sheet), with low energetic favorability for any interactions with residues of arm I. In our study, five of six reported key interacting residues contributed over 1.0 kcal/mol to the final binding energy for PPAR*γ* (Table 2): Cys285 (-5.6 kcal/mol), Ser289 (-1.9 kcal/mol), His323 (-2.6 kcal/mol), Tyr327 (-4.7 kcal/mol), and Tyr473(-1.1 kcal/mol). Though primary interaction was between residues Ile 341, Ser342, and Glu343 of the beta region (total 14.9 kcal/mol), and therefore occupying branch III of the binding pocket, two of three major interactions consistent with PPAR*γ* full agonists were achieved (His323 and Tyr473) which has been shown to be important for changing the protein conformation and recruiting the coactivator responsible for insulin sensitivity [44, 111]. As for PPAR*α*, the primary interaction was also between the conserved residues of the beta region, with an overall energy contribution of residues Val332, Ala333, and Tyr334 contributing a total of -16.5 kcal/mol to the total binding energy. Though this interaction in arm II provides evidence of the partial agonist activity, a weak binding interaction between His440 (analogous to His449 in PPAR*γ*) and chiglitazar was also achieved in this system. In the PPAR*α* system, chiglitazar has a similar binding mode as the dual agonist muraglitazar, forming two polar interactions with residues Gln277 and Ser280 on helix 3 (Figure 10(a)) in arm I, which are key residues responsible for agonist recognition [112]. Chiglitazar also formed additional pi-pi staking interactions with His440 on helix 11, formed hydrogen bonds with residue Thr279 on helix 3, and formed hydrophobic interactions with residue Leu460 and Tyr464 on helix 12 in arm I, which are important for stabilizing the AF-2 helix and maintaining the protein active conformation for recruiting the coactivator. In the PPAR*β* system, the most favorable binding interaction was with Lys331 of helix 7 followed by interaction with the beta region through residues Val305 and Ala306 contributing a total of -10.1 kcal/mol to the final binding energy. The PPAR*β* complex did not achieve any characteristic full agonist interactions, explaining the lower binding energy observed for this system. With all of this in mind, the ability of chiglitazar to activate PPAR*γ* was slightly different from other known PPAR*γ* agonists while still maintaining several key interactions that may be responsible for the decrease in the negative side effects observed in clinical trials.

Desmond's simulation interaction diagrams provided insight into the structural similarities and differences between systems. From the two-dimensional interaction diagrams, it was clear that the carbazole side chain of chiglitazar maintained hydrophobic interactions in each system. Specific attention is given to conserved residues Lys358, Lys331, and Lys367 for PPAR*α*, PPAR*β*, and PPAR*γ*, respectively. In both PPAR*α* and PPAR*γ*, this Lys residue contributes to the hydrophobic interactions surrounding the carbazole side chain. However, because of the difference in binding pose, the PPAR*β* complex shows Lys331 interacting with the oxygen at position 3 on chiglitazar. Comparing the interaction of Lys331 to the decomposition of MM-GBSA by residue, this interaction contributed the most to the overall energy (-9.3 kcal/mol) and may be the key residue involved in the binding of chiglitazar in the PPAR*β* complex system. From a visual inspection of the most abundant binding poses and by comparing the secondary structure elements, the 2′ helix was fundamentally different from both PPAR*α* and PPAR*γ* leaving free space around the binding pocket which appeared to limit the interactions of chiglitazar in the PPAR*β* complex system, which may explain the lower binding energy. The secondary structure elements of each trajectory is provided in [Supplementary-material supplementary-material-1] which shows PPAR*γ* having the greatest loss of helical structure at helix 12 over the course of the trajectory, followed by PPAR*α* and PPAR*β*. This we attribute to the increased fluctuations of helix 12 of PPAR*γ* when compared to PPAR*α* and PPAR*β*.

Other conserved residues involved in hydrophobic interactions were Val332, Val305, and Ile341 for the PPAR*α*, PPAR*β*, and PPAR*γ* receptors, respectively. The MM-GBSA binding energy decomposition by residue showed that the interactions of Val332 and Ile341 were the highest contributing residue for both the PPAR*α* (-8.7 kcal/mol) and PPAR*γ* (-9.6 kcal/mol) receptors, respectively, and the interaction of Val305 was the second highest contributor for the PPAR*β* system (-7.8 kcal/mol). This indicates the importance of this binding interaction for the activation of each PPAR*α*, PPAR*β*, and PPAR*γ*.

Further exploring the dynamics of each PPAR receptor, we used the combined trajectories of each system to calculate protein network models which identified connections between residues in the system (Figure 11(a)), generated a weighted representation of each connection (Figure 11(b)), and grouped each connection into communities based on stronger and more frequent connections to other nodes within those communities (Figure 12). From the weighted and unweighted network models, it was clear that the connections between the nodes of each system had distinct differences in both the connections to the node selected from the ligand as well as for helix 12. Furthermore, the community models showed entirely different communities for helix 12. In PPAR*α*, helix 12 has its own community with several critical edges linked to helix 3 and helix 11; in PPAR*β*, helix 12 is grouped in a community with the lower portion of helix 3; and in the PPAR*γ*, the model helix 12 is grouped with helix 11. The observed differences in connection between receptor subtypes could be linked to the reported activity of the receptor, where full agonists may activate the receptor through a direct interaction with helices 11 and 12, whereas the partial activation may be more linked to interactions with helices 3 and 4. For our PPAR*γ* system, our critical node analysis was consistent with the reported key residues for the full agonist's activation [109, 110]: H449, Cys285, Ser289, and Tyr327 [30, 69]. As for PPAR*α*, the critical node analysis accurately predicted the following known primary residues involved in partial activation: His440 (analogous to His449 in PPAR*γ*), Thr279 on helix 3, and Leu460 which is important for stabilizing the AF-2 helix and maintaining the protein active conformation for recruiting the coactivator.

**Figure 14:**
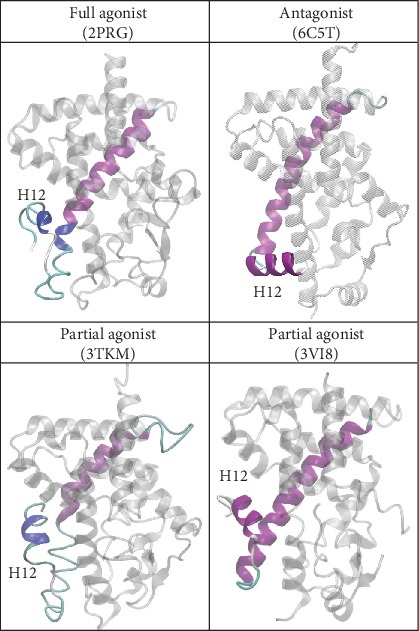
Proposed helix 12 conformations of the PPAR full agonist, partial agonists, and antagonist. Helices 10-11 are used as a references. Full and partial agonist conformations are derived from our simulations and antagonist conformation from the crystal structure. Coloring of helices 10-12 is based on secondary structure.

To further probe the overall motion of the receptors, we performed a principal component analysis based on the combined trajectories. Through analysis of the PCA ([Supplementary-material supplementary-material-1]) and the RMSF ([Supplementary-material supplementary-material-1]) of each system, the top five modes provided insight that is consistent with an ongoing hypothesis into the activity of the PPAR receptors. In short, it is hypothesized that the conformation of helix 12 is determined by the activity of the ligand (i.e., agonist and antagonist) [106]. Building from that, our observations have led us to speculate that rather than being bent fully in the direction of the agonist versus antagonist conformation, a partial agonist can adopt a more linear conformation; we present a visual example of this in Figure 14. In addition to the overall conformation of helix 12, we also speculate that the degree of flexibility plays a role in activity.

**Figure 15:**
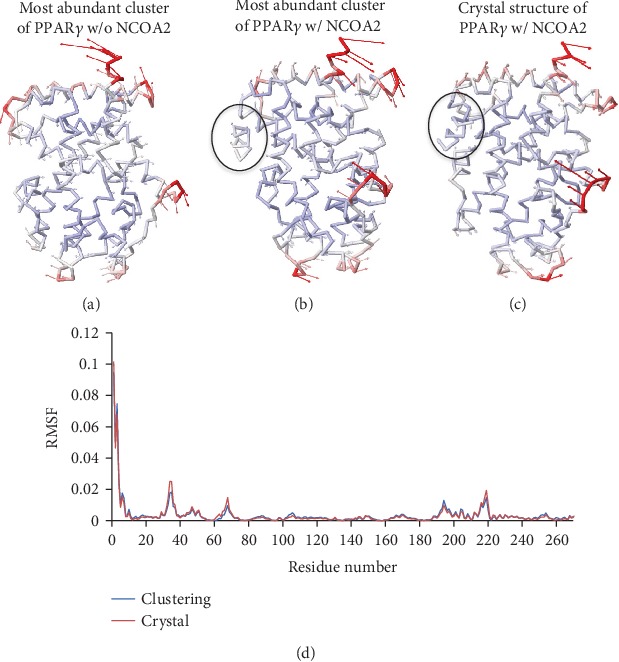
Anisotropic network models of our most abundant cluster of PPAR*γ* (a), our most abundant cluster of PPAR*γ* with nuclear receptor coactivator 2 (NCOA2) peptide docked (b), and the PPAR*γ*-NCOA2 complex from the original crystal structure (PDB ID: 3DZY) (c), plus an RMSF plot (d) comparing the fluctuations of the PPAR*γ*-NCOA2 complex derived from docking NCOA2 into our most abundant cluster of PPAR*γ* (b; blue) to the original crystal structure of the PPAR*γ*-NCOA2 complex (c; red).

To gain more insight into the activation mechanism, we used a coarse-grained anisotropic network model to calculate the normal modes on PPAR*γ* with the nuclear receptor coactivator 2 (NCOA2) peptide docked. NCOA2 is a key part of the full structure of the PPAR*γ*-retinoid X receptor (RXR) alpha complex on DNA ([Supplementary-material supplementary-material-1]), so we found it important to understand its role by exploring the top vibrational mode (Figure 15). From our analysis, it is clear that the directionality of H12 is switched in the presence of NCOA2 which we speculate may play a role in the complex that each PPAR subtype forms with both the DNA binding domain (DBD) and the retinoid X receptor (RXR) at that site. In mode 1 of PPAR*γ*, we observed that H12 was extremely flexible, folding left of the front view point into a conformation that opens up the coactivator binding site between helix 12 and helix 4/5. Although PPAR*α* is also in a left bent conformation, the flexibility of helix 12 itself is minimal. In this case, helix 12 remains stable and the C-terminal region is more flexible in a stretching manner. Evident from the RMSF (Figure 15(d)) of the lowest energy mode of the PPAR*γ*-NCOA2 complex derived from docking NCOA2 into our most abundant cluster of PPAR*γ* and the complex of the original crystal structure of the PPAR*γ*-NCOA2, our MD-derived system produces nearly identical RMSF when compared to the crystal structure. The closely comparable RMSF results not only suggest the accuracy of this prediction but reinforce the accuracy of the computational methods used for our simulations.

Extrapolating this data with comparison to our principal component analysis, the position of helix 12 in the PPAR*α* system may help to explain PPAR*α*'s increased binding energy over PPAR*β*, whereas the lack of flexibility may explain PPAR*α*'s lower binding energy when compared to PPAR*γ*. PPAR*β*, on the other hand, is somewhere between the two extremes, the difference being the direction which helix 12 is moving. Rather than folding to the left, helix 12 adopts a more linear conformation, significantly reducing the area of the coactivator binding pocket when compared to PPAR*γ*. These observations are supported by the RMSF of both the original trajectory (Figure 7) as well as the RMSF of mode 1 from the normal mode analysis (Figure 13), where the fluctuations are largest in PPAR*γ* followed by PPAR*β* and PPAR*α*.

Further support of our helix 12 hypothesis is provided through a deeper evaluation of the RMSD of helix 12 in each system (Figures [Supplementary-material supplementary-material-1]). We measured the RMSD of helix 12 in each system using the initial crystal structure as a reference and defined helix 12 residues for PPAR*α* (448-468), PPAR*β* (421-441), and PPAR*γ* (457-477) using the flexible portion of this region ([Supplementary-material supplementary-material-1]). From this, we observed a wide range of RMSD's for PPAR*γ* when compared to PPAR*α* and PPAR*β*. Specifically, we saw that PPAR*γ* had two major RMSD's of 2.5 Å and 4.5 Å where PPAR*α* was primarily 1.5 Å and PPAR*β* was primarily 2.5 Å. We have also included a time series of each of the three trajectories of RMSD per system and structural representation of the conformation of H12 in the two most abundant RMSDs (2.5 and 4.5 Å) for PPAR*γ* ([Supplementary-material supplementary-material-1]). The difference in the helix 12 RMSD for PPAR*γ* when compared to PPAR*α* and PPAR*β* further supports our hypothesis that the degree of flexibility at helix 12 plays a role in an activity.

Although the omega loop between helix 2′ and 3 is also hypothesized to be involved in the allosteric activation of the receptor, the receptor structures used in our study have small breaks in the omega loop sequence, as a result of a structural alignment. Therefore, we are unable to provide insight into how this portion of the receptor is linked to its biological activity. In order to do this, a homology model of the receptors would need to be built to fill in the gaps in sequence and then used in a new set of simulations, which is a direction we are likely to pursue in a future study.

With the detailed interaction profile provided in this study, the key residues as well as their major poses were identified and may be useful in designing other partial or selective PPAR agonists with an enhanced binding profile by activating only the key portion of the receptors, or by using the key interacting residue information to modulate the interactions with each receptor subtype. This may ultimately help to identify a new medication which completely eliminates negative side effects associated with current T2DM medications and provide a higher quality of life from those being treated for T2DM.

## 5. Conclusions

With type 2 diabetes mellitus (T2DM) affecting such a broad range of the population, there is a dire need for effective treatment with minimal side effects. Previous T2DM medications like rosiglitazone (Avandia) and pioglitazone (Actos) are thiazolidinediones (TZDs) that are insulin sensitizers acting as full agonists to the PPAR*γ* receptor. TZDs worked efficiently to reduce antihyperlipidemic and antihypertensive effects; however, with higher chances of myocardial infarction and weight gain, amongst other negative side effects, use has been significantly restricted to a last line of defense against diabetes. Chiglitazar is a new generation of non-TZD T2DM medications able to regulate gene expression due to its configuration-restricted binding as well as the phosphorylation inhibition of hPPAR*γ* with a significantly lower chance of cardiac toxicity when compared to TZDs. Though initially thought to be a dual agonist of PPAR*α* and PPAR*γ*, research over the past decade has provided evidence of chiglitazar's pan-agonist activity toward each of the PPAR receptor subtypes. In this study, we used molecular dynamics (MD) simulation and a MM-GBSA binding energy analysis to elucidate the mechanism driving the interaction of chiglitazar and the PPAR receptor subtypes PPAR*α*, PPAR*β*, and PPAR*γ*. Our MM-GBSA binding energy calculation revealed that chiglitazar most favorably bound to hPPAR*γ* (-144.6 kcal/mol) followed by hPPAR*α* (-138.0 kcal/mol) and hPPAR*β* (-131.2 kcal/mol). Through visual inspection of the structural clustering analysis, decomposition of the MM-GBSA binding energy by residue, and by the use of two-dimensional interaction diagrams, key residues involved in the binding of chiglitazar were identified and characterized for each complex system. Our detailed analysis supports chiglitazar's activity as a pan-agonist and provides dynamic details to describe the underlying mechanism used to fully activate PPAR*γ* and partially activate PPAR*α* and PPAR*β*, which may aid in further development of this new generation of medication. Our detailed analyses support that the conformation and dynamics of helix 12 play a critical role in determining the different activities of the different types of ligands (e.g., full agonist vs. partial agonist). Rather than being bent fully in the direction of the agonist versus antagonist conformation, a partial agonist can induce a more linear conformation and have a lower degree of flexibility.

## Figures and Tables

**Table 1:** MM-GBSA binding energies with standard deviation of chiglitazar bound to PPAR*α*, PPAR*β*, and PPAR*γ* receptors.

Term	PPAR*α*	PPAR*β*	PPAR*γ*
Δ*E* (kcal/mol)	-138.0 ± 7.3	−135.9 ± 5.3	−144.6 ± 5.6
ΔΔ*E* (kcal/mol)	6.6	8.7	0.0
ΔVDW	−82.9 ± 3.8	−76.4 ± 2.9	−87.9 ± 3.0
ΔΔVDW	5.0	11.5	0.0
ΔLIPO	−67.5 ± 3.0	−71.9 ± 2.4	−71.3 ± 2.2
ΔGBELE	12.4 ± 4.8	12.4 ± 2.3	14.6 ± 3.5
Experimental EC50 (*μ*M)	1.2 ± 0.3	1.7 ± 0.2	0.08 ± 0.02

Δ*E*: MM-GBSA binding energy (complex-receptor-ligand). ΔΔ*E*: relative binding energy with reference to an active complex. ΔVDW: change of van der Waals energy (VDW+*π*-*π* stacking+self-contact correction) in gas phase upon complex formation. ΔGBELE: change of electrostatic interactions (GB/generalized born electrostatic solvation energy+ELE/Coulomb energy+hydrogen bonding) upon complex formation. Change of lipophilic term (lipophilic energy) upon complex formation.

**Table 2:** Key residues of each receptor interaction with chiglitazar.

PPAR*α* (kcal/mol)		PPAR*β* (kcal/mol)		PPAR*γ* (kcal/mol)	
ILE_241	-1.2				
LEU_254	-1.5			ILE_262	-2.7
VAL_255	-2.9				
LEU_258	-2.9				
		VAL_245	-1.0	ILE_281	-1.1
PHE_273	-1.0	PHE_246	-2.4	PHE_282	-1.2
CYS_275	-2.6	ARG_248	-5.3	GLY_284	-2.6
CYS_276	-6.7	CYS_249	-4.9	CYS_285	-5.6
GLN_277	-1.4	GLN_250	-1.9	GLN_286	-3.8
				PHE_287	-3.2
THR_279	-4.7	THR_252	-4.2	ARG_288	-5.1
SER_280	-3.2			SER_289	-1.9
THR_283	-3.0				
				HIS_323	-2.6
ILE_317	-2.9	ILE_290	-1.6	ILE_326	-4.3
PHE_318	-1.8	PHE_291	-1.9	TYR_327	-4.7
				MET_329	-1.1
LEU_321	-5.0	LEU_294	-4.7	LEU_330	-4.3
		ILE_297	-2.6	LEU_333	-2.4
		VAL_298	-1.4		
MET_330	-1.8	LEU_303	-4.2		
VAL_332	-8.7	VAL_305	-7.8	ILE_341	-9.6
ALA_333	-4.2	ALA_306	-2.3	SER_342	-4.3
TYR_334	-3.6			GLU_343	-1.0
ILE_339	-1.2			MET_348	-1.9
ILE_354	-1.5	ILE_327	-1.6	PHE_363	-1.0
MET_355	-1.8				
LYS_358	-3.6	LYS_331	-9.3	LYS_367	-1.2
HIS_440	-1.6				
		MET_416	-3.1		
VAL_444	-1.0				
		ILE_420	-1.2		
		LEU_429	-1.2	LEU_465	-1.7
				LEU_469	-2.1
				TYR_473	-1.1

## Data Availability

The structure data used to support the findings of this study are available from the corresponding author upon request.

## Supplementary Materials

Supplementary Materials Table S1: PDB IDs of known crystal ligands and activity toward each of the PPAR*α*, PPAR*β*, and PPAR*γ* receptors. Table S2: root mean square fluctuations of each helix of the PPAR subtypes. Table S3: list of critical residues identified from the network analysis. Table S4: comparison of the MM-GBSA binding energies of our MD-simulated crystal complexes to the MD-simulated PPAR*α*, PPAR*β*, and PPAR*γ* receptors in complex with chiglitazar. Figure S1: full structure of the PPAR*γ-* (silver) retinoid X receptor (RXR) alpha (blue) complex on DNA (black) with rosiglitazone (yellow) and nuclear receptor coactivator 2 (NCOA2) (green) (PDB ID: 3DZY). Figure S2: protein and ligand RMSD for the simulated crystal structure systems for PPAR*α* (PDB ID: 3VI8), PPAR*β* (PDB ID: 3TKM), and PPAR*γ* (PDB ID: 2PRG). Figure S3: comparison of the induced fit docking, MD-derived complexes, and the superimposed complexes PPAR*α* (PDB ID: 3VI8), PPAR*β* (PDB ID: 3TKM), and PPAR*γ* (PDB ID: 2PRG). Figure S4: representative structures and abundance of the top structural families from the clustering analysis of the combined trajectories. Figure S5: average 2D interaction profile of chiglitazar in complex with PPAR*α*, PPAR*β*, and PPAR*γ* of the multiple trajectory runs: a histogram of protein-ligand interactions. Figure S6: average ligand torsion (dihedral angle) profile of chiglitazar in complex with PPAR*α*, PPAR*β*, and PPAR*γ* from the combined trajectory runs. Figure S7: the top five modes (1-5) of the trajectory-based principal component analysis performed using VMD's Normal Mode Wizard for the combined blocks of the trajectories for PPAR*α* (A), PPAR*β* (B), and PPAR*γ* (C). The color scheme is as follows: blue—low movement; grey—moderate movement; red—maximum movement. Vectors of 3.5 Å or greater are shown and represent the directionality of movement where larger vectors represent greater fluctuations. Figure S8: RMSF of the top 5 normal modes of the trajectories, derived from VMD's Normal Mode Wizard. Figure S9: docking pose (A) and 2D interaction diagram (B) of chiglitazar in complex with PPAR*γ* (from PDB ID: 2PRG). Figure S10: secondary structure element timelines for each of the three trajectories of PPAR*α* (A), PPAR*β* (B), and PPAR*γ* (C). Figure S11: position of helix 12 (red) over the course of the combined trajectory including a histogram showing the RMSD distribution of helix 12 as well as the time series of the helix 12 RMSD for each trajectory (trajectory 1—blue; trajectory 2—red; trajectory 3—green) for PPAR*α* (A), PPAR*β* (B), and PPAR*γ* (C). C-terminal is represented as a blue ball. Figure S12: two most abundant conformations of helix 12 based on RMSD. Superimposition shows the conformation of helix 12 at 2.5 Å RMSD in blue and at 4.5 Å RMSD in red.
