# Supplementary material for: To Probe Full and Partial Activation of Human Peroxisome Proliferator-Activated Receptors by Pan-Agonist Chiglitazar Using Molecular Dynamics Simulations
Source: PPAR Res. 2020 Apr 1;2020:5314187. doi: 10.1155/2020/5314187 (PMC7152983; doi:10.1155/2020/5314187)
Supplement: Supplementary Materials — Table S1: PDB IDs of known crystal ligands and activity toward each of the PPARα, PPARβ, and PPARγ receptors. Table S2: root mean square fluctuations of each helix of the PPAR subtypes. Table S3: list of critical residues identified from the network analysis. Table S4: comparison of the MM-GBSA binding energies of our MD-simulated crystal complexes to the MD-simulated PPARα, PPARβ, and PPARγ receptors in complex with chiglitazar. Figure S1: full structure of the PPARγ- (silver) retinoid X receptor (RXR) alpha (blue) complex on DNA (black) with rosiglitazone (yellow) and nuclear receptor coactivator 2 (NCOA2) (green) (PDB ID: 3DZY). Figure S2: protein and ligand RMSD for the simulated crystal structure systems for PPARα (PDB ID: 3VI8), PPARβ (PDB ID: 3TKM), and PPARγ (PDB ID: 2PRG). Figure S3: comparison of the induced fit docking, MD-derived complexes, and the superimposed complexes PPARα (PDB ID: 3VI8), PPARβ (PDB ID: 3TKM), and PPARγ (PDB ID: 2PRG). Figure S4: representative structures and abundance of the top structural families from the clustering analysis of the combined trajectories. Figure S5: average 2D interaction profile of chiglitazar in complex with PPARα, PPARβ, and PPARγ of the multiple trajectory runs: a histogram of protein-ligand interactions. Figure S6: average ligand torsion (dihedral angle) profile of chiglitazar in complex with PPARα, PPARβ, and PPARγ from the combined trajectory runs. Figure S7: the top five modes (1-5) of the trajectory-based principal component analysis performed using VMD's Normal Mode Wizard for the combined blocks of the trajectories for PPARα (A), PPARβ (B), and PPARγ (C). The color scheme is as follows: blue—low movement; grey—moderate movement; red—maximum movement. Vectors of 3.5 Å or greater are shown and represent the directionality of movement where larger vectors represent greater fluctuations. Figure S8: RMSF of the top 5 normal modes of the trajectories, derived from VMD's Normal Mode Wizard. Figure S9: docking p [file 5314187.f1.docx]

**To probe full and partial activation of human Peroxisome Proliferator-Activated Receptors by pan-agonist Chiglitazar using molecular dynamics simulations**

Holli-Joi Sullivan†^1^, Xiaoyan Wang,^†2,3^ Shaina Nogle,^†1^ Siyan Liao^1, 4^, and Chun Wu*^1^

**Table S1** PDB IDs of known crystal ligand and activity toward each the PPARα, PPARβ, and PPARγ receptors.

| Crystal Ligands | PDB | Activity |
| --- | --- | --- |
| **PPARα** | 3VI8 | Partial Agonist (APHM13) |
| **PPARβ** | 3TKM | Partial Agonist (GW0742) |
| **PPARγ** | 2PRG | Full Agonist (Rosiglitazone) |

**Table S2**. Root mean square fluctuations of each helix of the PPAR subtypes.

| **Helix** | **PPARα** | **PPARβ** | **PPARγ** |
| --- | --- | --- | --- |
| 1 | 1.309267 | 0.905778 | 0.96625 |
| 2 | 3.5285 | 1.2925 | 1.429 |
| 2' | 1.8215 | 2.506 | 1.80475 |
| 3 | 0.900167 | 0.7105 | 1.109308 |
| 4 | 1.344143 | 0.822083 | 1.174375 |
| 5 | 0.955889 | 1.19575 | 1.070917 |
| 6 | 0.790667 | 0.8648 | 1.2525 |
| 7 | 1.1465 | 1.0023 | 1.241824 |
| 8 | 0.76275 | 0.605 | 0.667083 |
| 9 | 1.251045 | 0.897773 | 0.859111 |
| 10 | 0.97075 | 0.977333 | 1.085813 |
| 11 | 1.016261 | 1.674923 | 1.539313 |
| 12 | 1.3432 | 1.525 | 1.8386 |

**Table S3**. List of critical residues identified from the network analysis.

| **Critical Residue Identification** | | |
| --- | --- | --- |
| **PPARα** | **PPARβ** | **PPARγ** |
| Tyr211 | Gly167 | Phe226 |
| Tyr214 | Leu187 | Leu228 |
| Asn217 | Asn191 | Lys232 |
| Asn219 | Thr193 | Ala235 |
| Ile241 | Lys194 | Ala278 |
| Leu247 | Lys195 | Ile281 |
| Ile272 | Lys196 | Cys285 |
| Thr279 | Arg198 | Arg288 |
| Thr283 | Leu201 | Ser289 |
| Phe290 | His214 | Glu324 |
| Lys310 | Leu219 | Ile325 |
| Val313 | Trp220 | Ile326 |
| Met320 | Cys249 | Tyr327 |
| Ser322 | Thr252 | Thr328 |
| Ile339 | Val257 | Met329 |
| Glu369 | Glu259 | Ala331 |
| Asp371 | Gln278 | Leu333 |
| Ser373 | Gly285 | Met334 |
| Val379 | His287 | Val339 |
| Ala380 | Glu288 | Met348 |
| Ile382 | Ala289 | Phe360 |
| Met400 | Ile290 | Phe363 |
| Ile404 | Phe291 | Phe374 |
| Lys429 | Met293 | Leu377 |
| Leu433 | Leu294 | Asp383 |
| Arg434 | Asn299 | Leu384 |
| Val437 | Leu304 | Ala385 |
| Thr438 | Val312 | Ile386 |
| His440 | Phe316 | Phe387 |
| Ala441 | Ser319 | Ile388 |
| His457 | Leu320 | Glu407 |
| Leu459 | Lys322 | Gln410 |
| Leu460 | Lys337 | Asp411 |
| Glu462 | Asn339 | Asn412 |
| Ile463 | Leu341 | Leu414 |
|  | Asp345 | Leu417 |
|  | Val367 | Gln420 |
|  | Pro368 | Asn424 |
|  | Arg369 | Leu435 |
|  | Glu371 | Lys438 |
|  | Ala372 | Met439 |
|  | Leu395 | Leu442 |
|  | Pro397 | Val446 |
|  | Lys398 | His449 |
|  | Gln401 |  |
|  | Lys402 |  |
|  | Met403 |  |
|  | Asp405 |  |
|  | Leu409 |  |
|  | Met416 |  |
|  | His430 |  |
|  | Leu432 |  |

**Table S4.** Comparison of the MM-GBSA binding energy of our MD simulated crystal complexes to the MD simulated PPARα, PPARβ, and PPARγ receptors in complex with Chiglitazar^1,2^.

| System | ΔE | ΔVDW | ΔLIPO | ΔGBELE |
| --- | --- | --- | --- | --- |
| PPARα-Chi | -138.0 ±7.3 | -82.9 ± 3.8 | -67.5 ± 3.0 | 12.4 ± 4.8 |
| Crystal PPARα | -125.8±8.8 | -67.7±3.8 | -68.1±3.3 | 10.0±4.8 |
| PPARα ΔΔE | **-12.2** | **-15.2** | **0.6** | **-2.4** |
| PPARβ-Chi | -135.9± 5.3 | -76.4 ± 2.9 | -71.9 ± 2.4 | 12.4 ± 2.3 |
| Crystal PPARβ | -123.3±6.2 | -56.8±3.24 | -59.5±2.60 | -6.9±2.61 |
| PPARβ ΔΔE | **-12.6** | **-19.53** | **-12.4** | **19.3** |
| PPARγ-Chi | -144.6±5.6 | -87.9 ± 3.0 | -71.3 ± 2.2 | 14.6 ± 3.5 |
| Crystal PPARγ | -100.9±4.1 | -55.7±2.53 | -48.1±1.95 | 2.8±3.42 |
| PPARγ ΔΔE | **-43.6** | **-32.2** | **-23.2** | **11.8** |

^1^Binding energy reported in kcal/mol.

^2^See Table 1 and methods section for full explanation of terms.


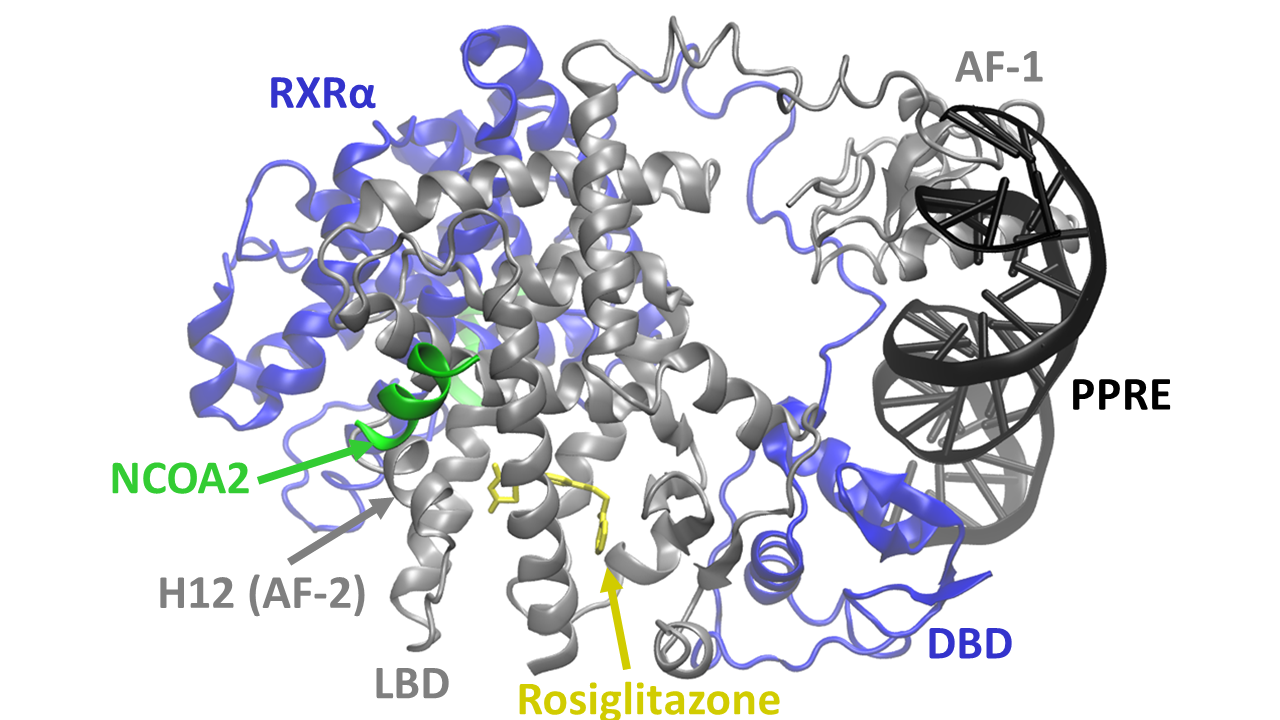


**Figure S1.** Full structure of the PPARγ (silver)-Retinoid X Receptor (RXR) alpha (blue) complex on DNA (black) with Rosiglitazone (yellow) and Nuclear Receptor Coactivator 2 (NCOA2) (green) (PDB ID: 3DZY).

| 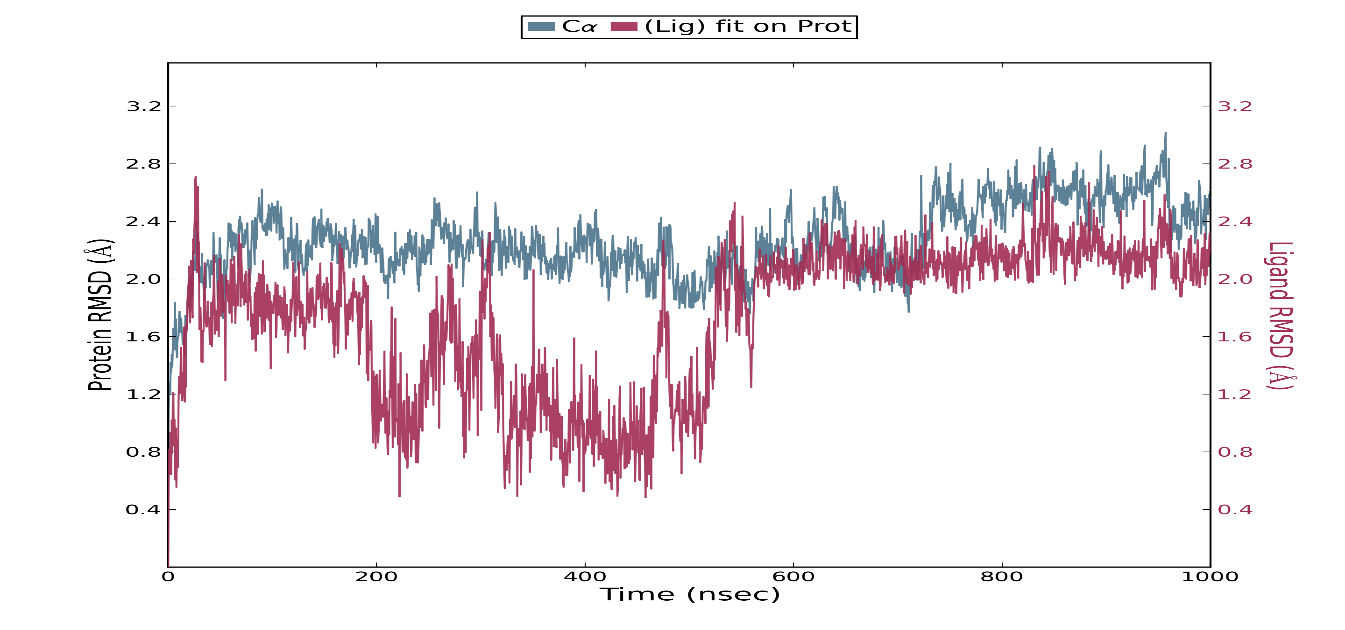  α |
| --- |
| β 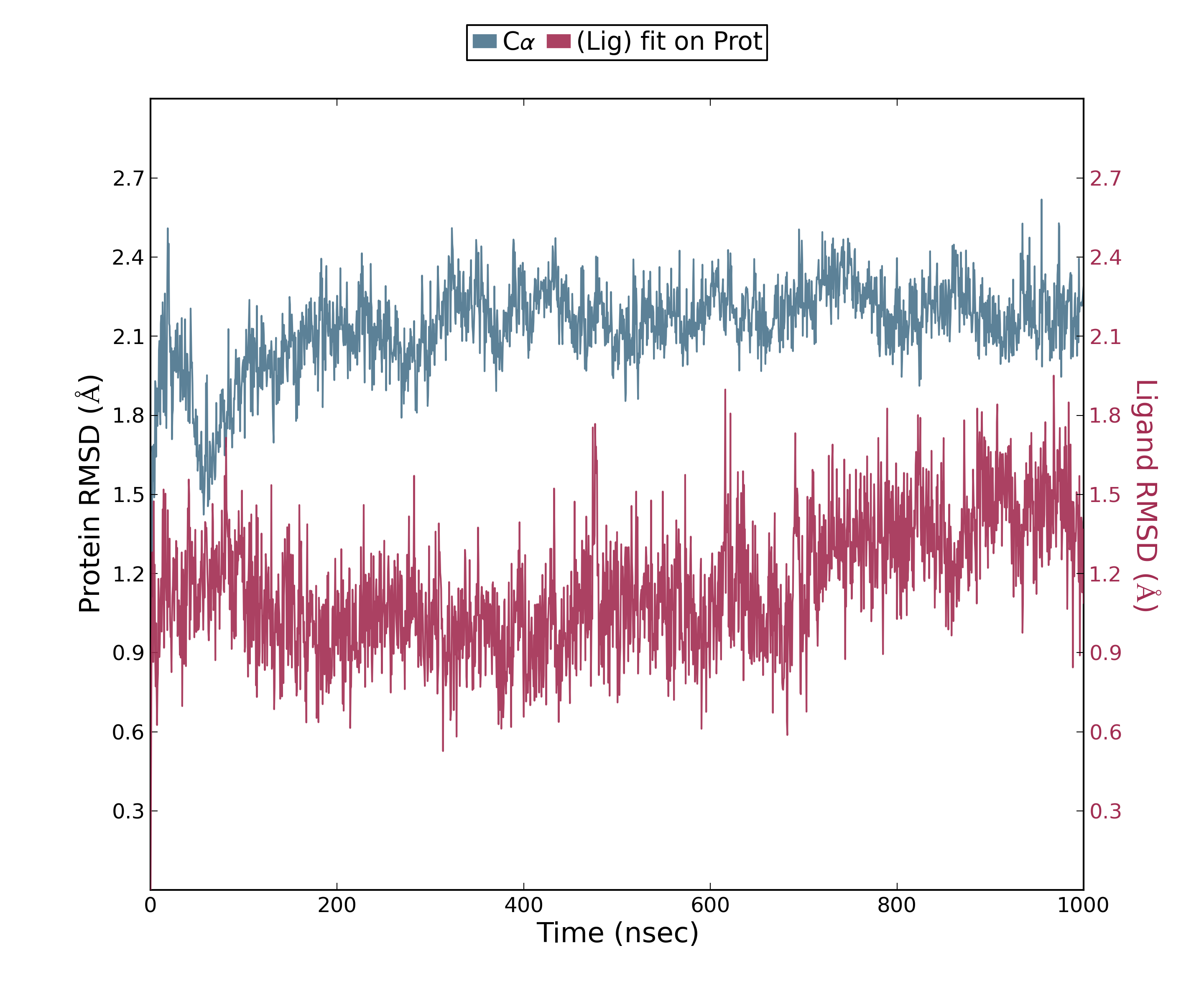 |
| 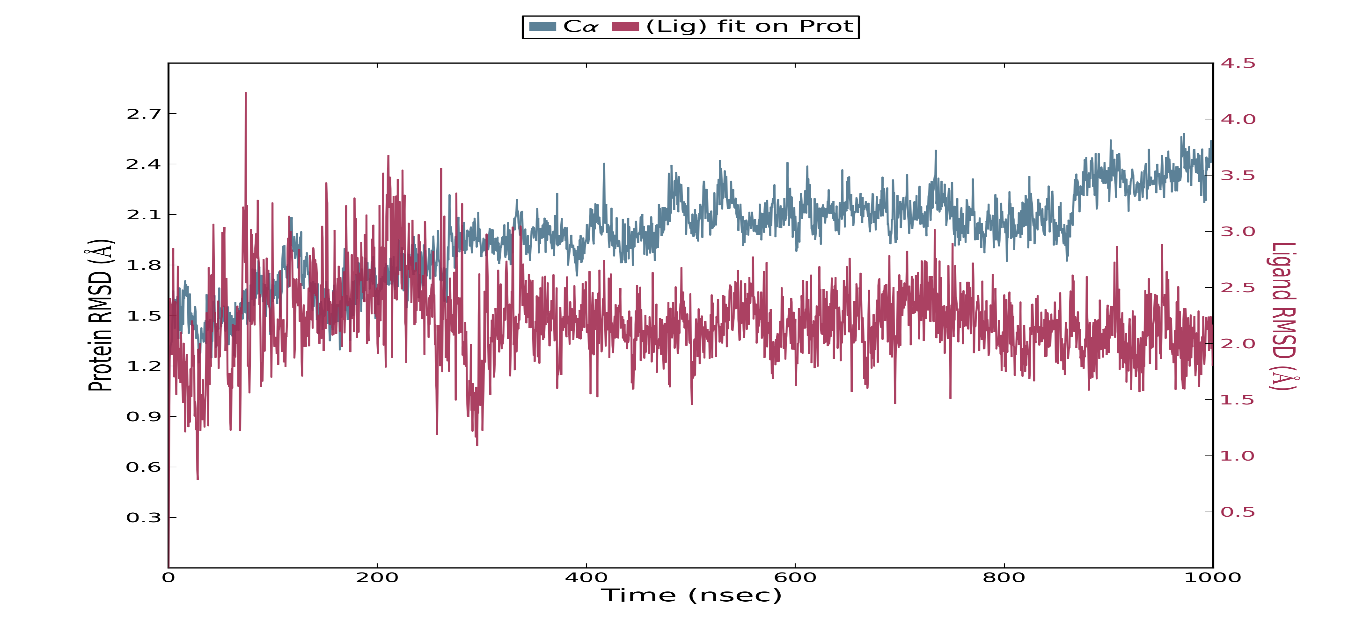  γ |

**Figure S2.** Protein and ligand RMSD for the simulated crystal structure systems for PPARα (PDB ID: 3VI8), PPARβ (PDB ID: 3TKM), and PPARγ (PDB ID: 2PRG).

| IFD | MD | SUPERIMPOSED | LIGAND |
| --- | --- | --- | --- |
| 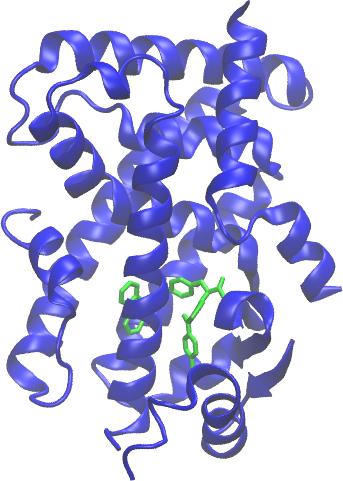 | 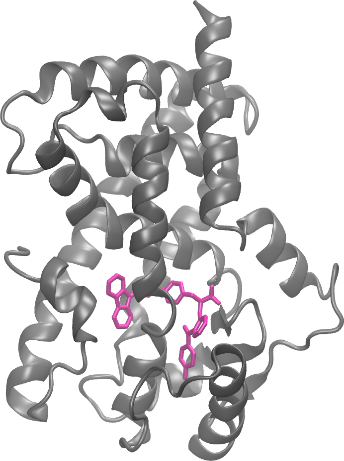 | 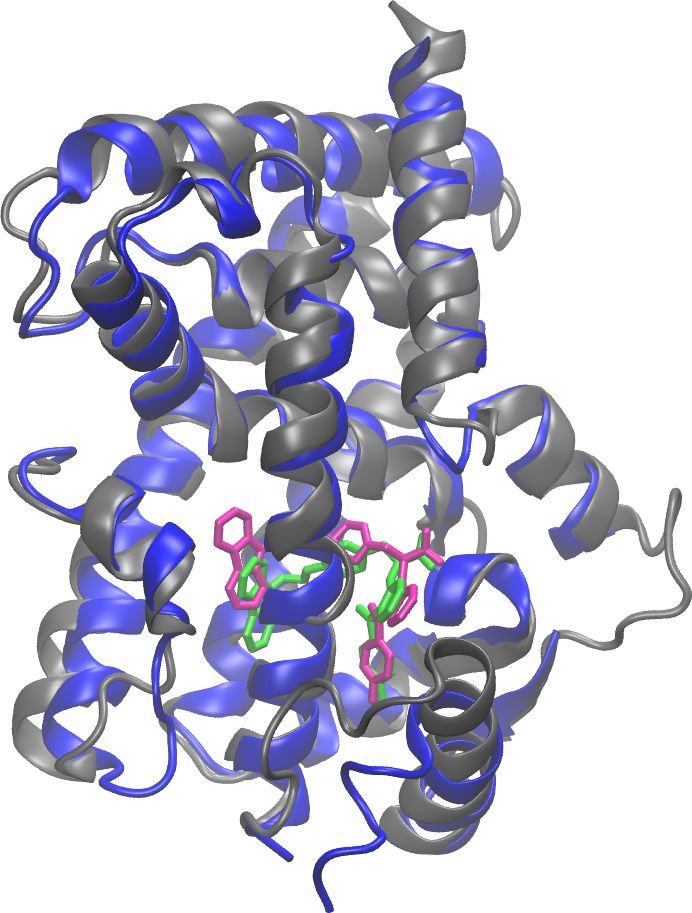 | 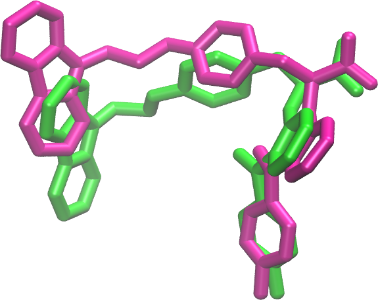 |
| 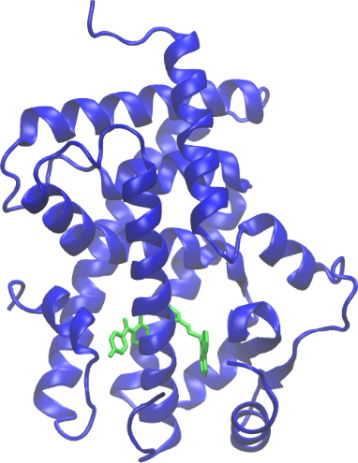 | 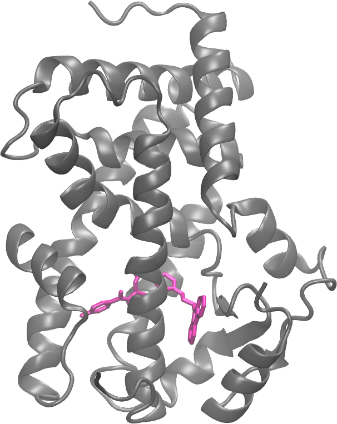 | 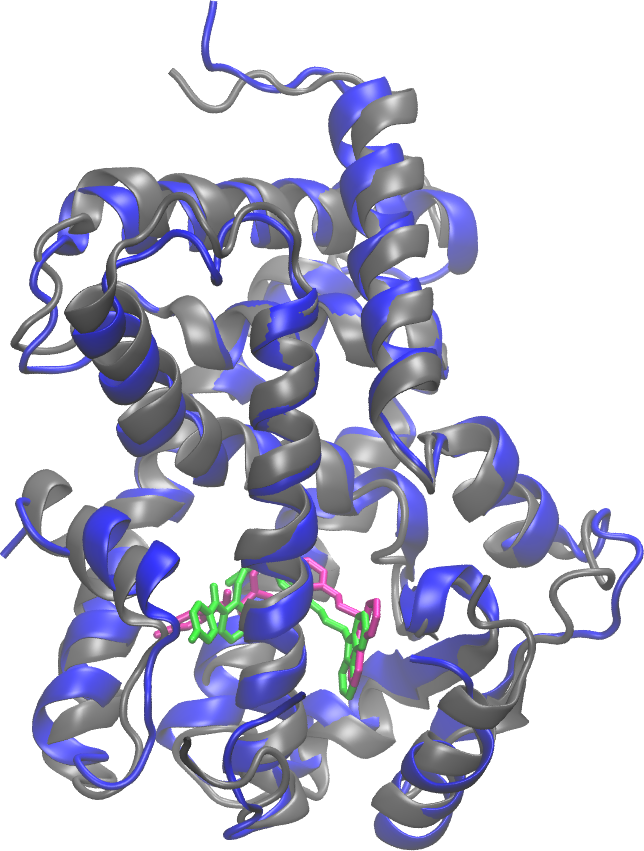 | 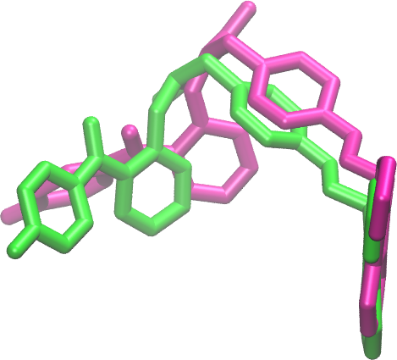 |
| 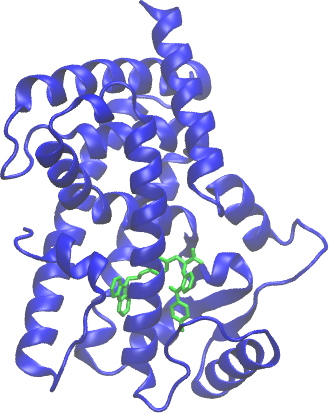 | 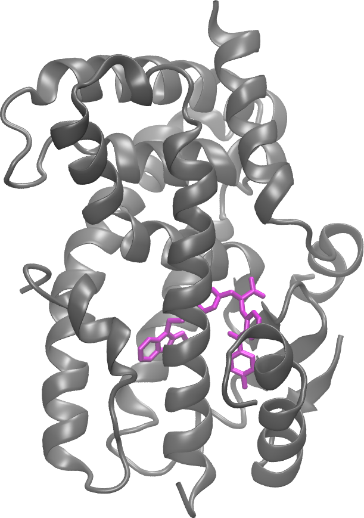 | 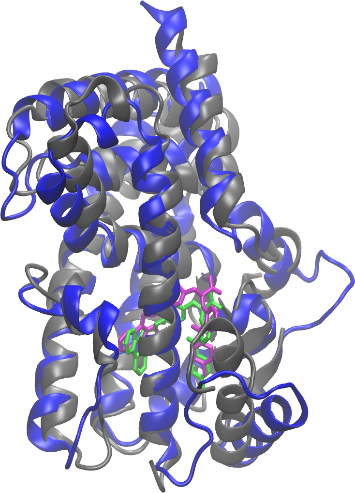 | 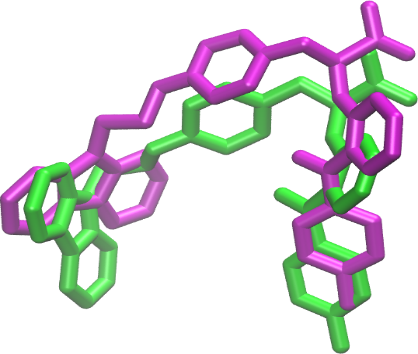 |

**Figure S3.** Comparison of the Induced fit docking, MD derived complexes, and the superimposed complexes PPARα(PDB ID: 3VI8), PPARβ(PDB ID: 3TKM), and PPARγ(PDB ID: 2PRG).

| PPARα | 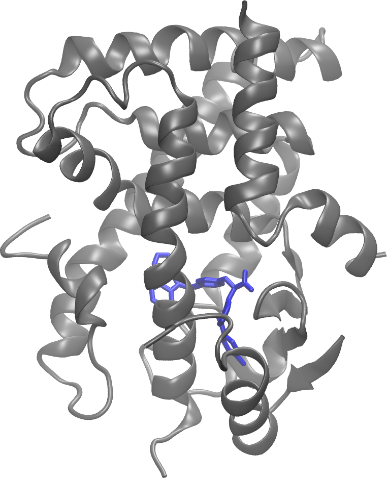 | 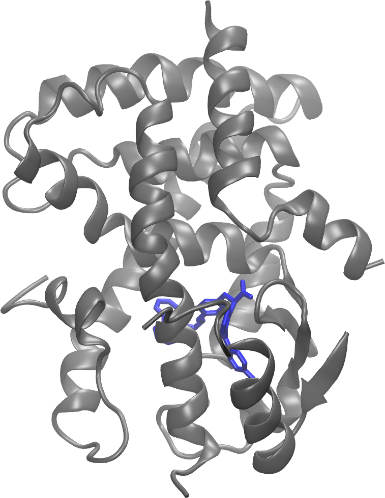 | 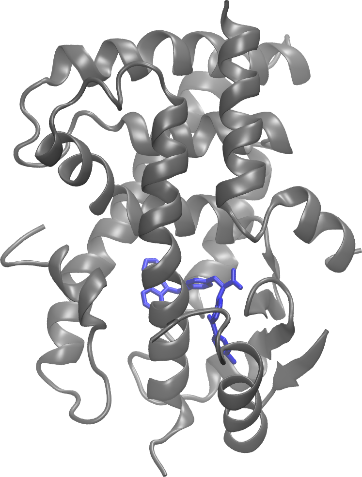 |
| --- | --- | --- | --- |
|  | 48.9% | 31.9% | 18.1% |
| PPARβ | 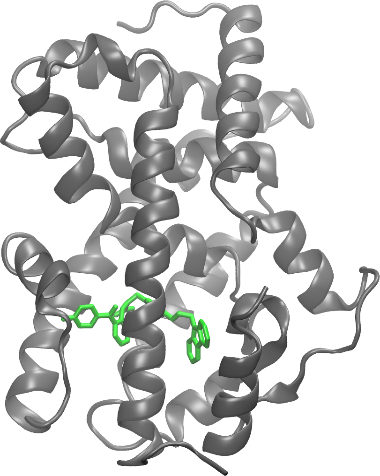 | 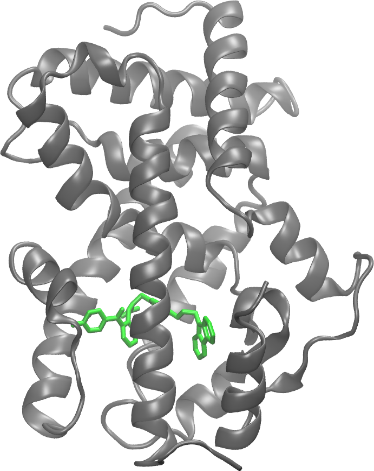 |  |
|  | 98.7% | 1.09% |  |
| PPARγ | 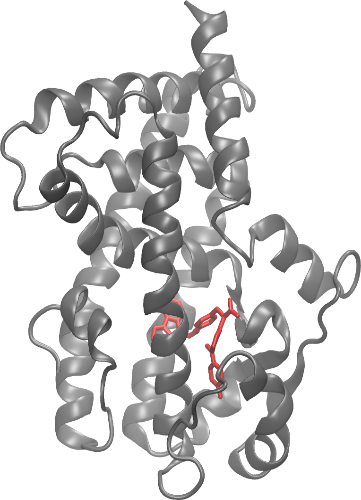 |  |  |
|  | 100% |  |  |

**Figure S4**. Representative structures and abundance of the top structural families from the clustering analysis of the combined trajectories.

| 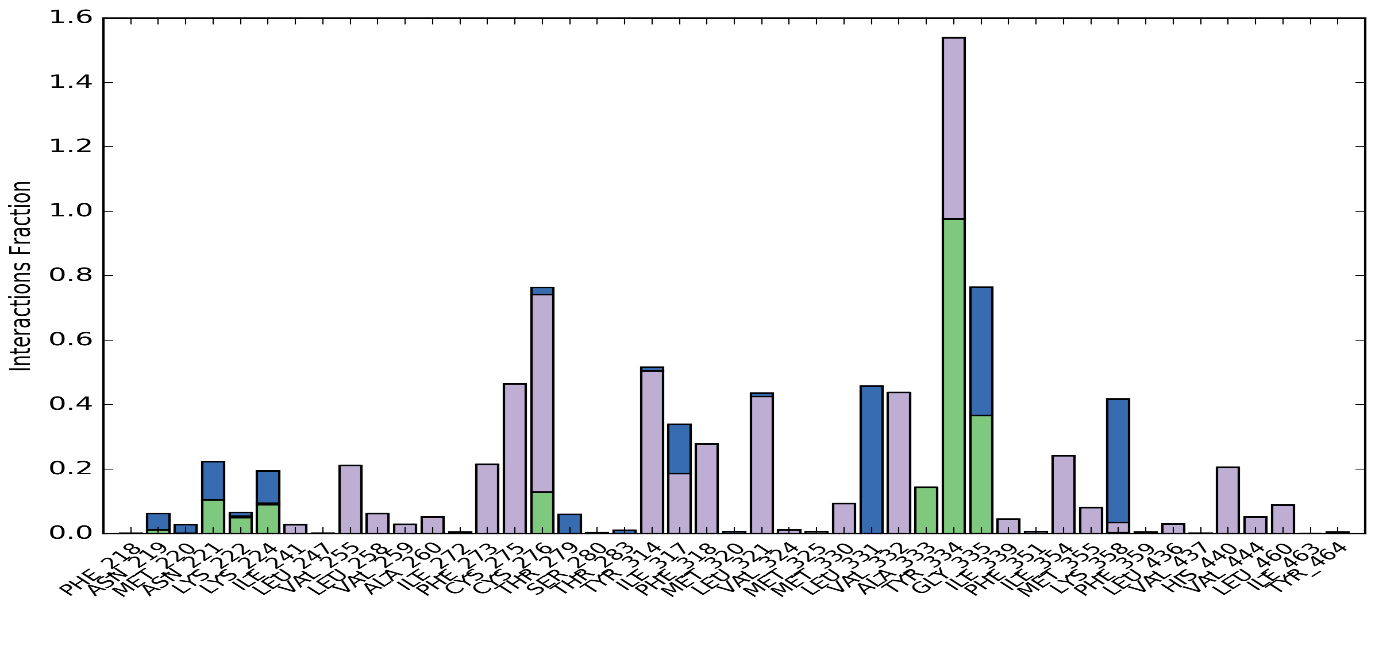  PPARα |
| --- |
|  |
| 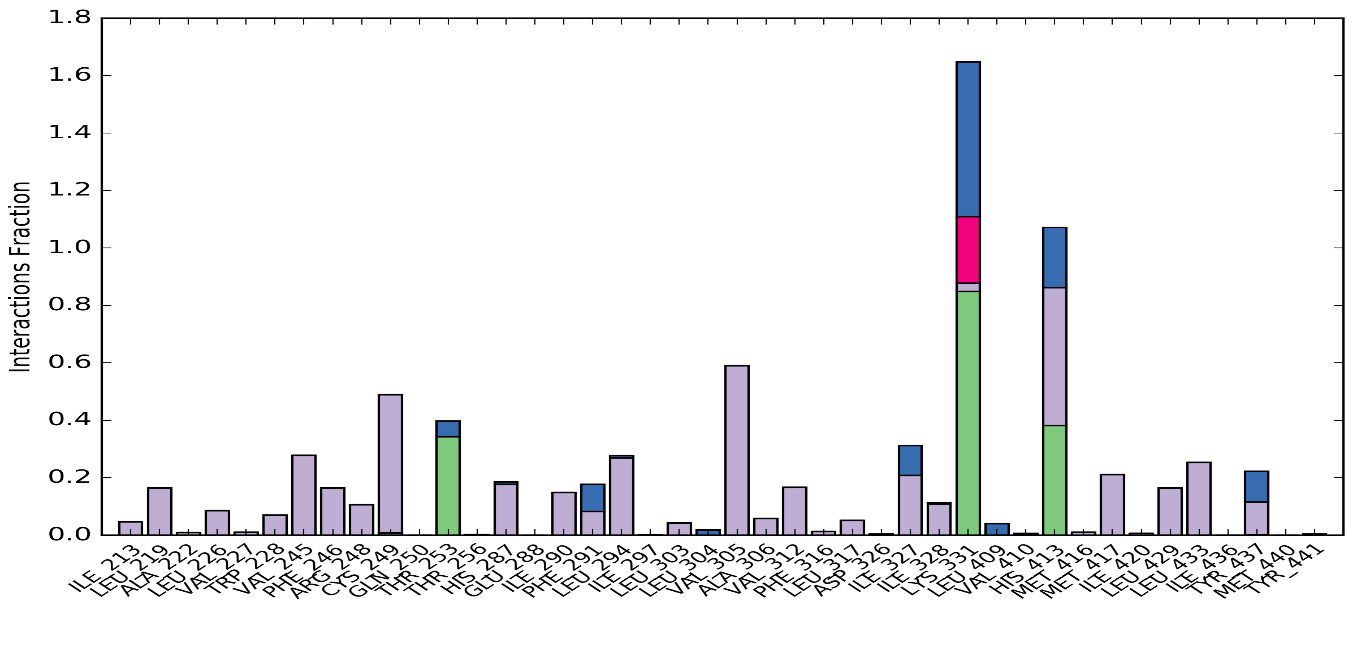  PPARβ |
|  |
| 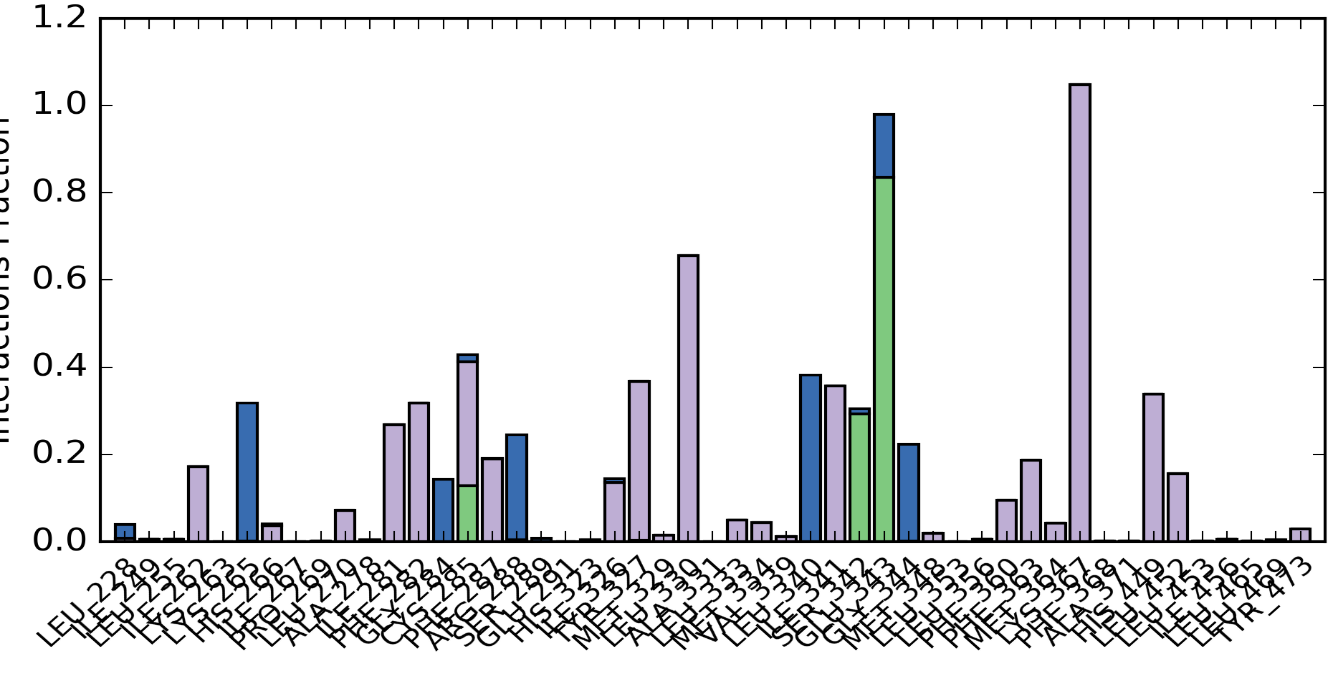  PPARγ |
|  |
| 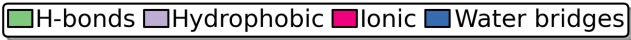 |

**Figure S5.** Average 2D interaction profile of chiglitazar in complex with PPARα, PPARβ, and PPARγ of the multiple trajectory runs: a histogram of protein ligand interactions.

**Dihedral angle distribution**

| 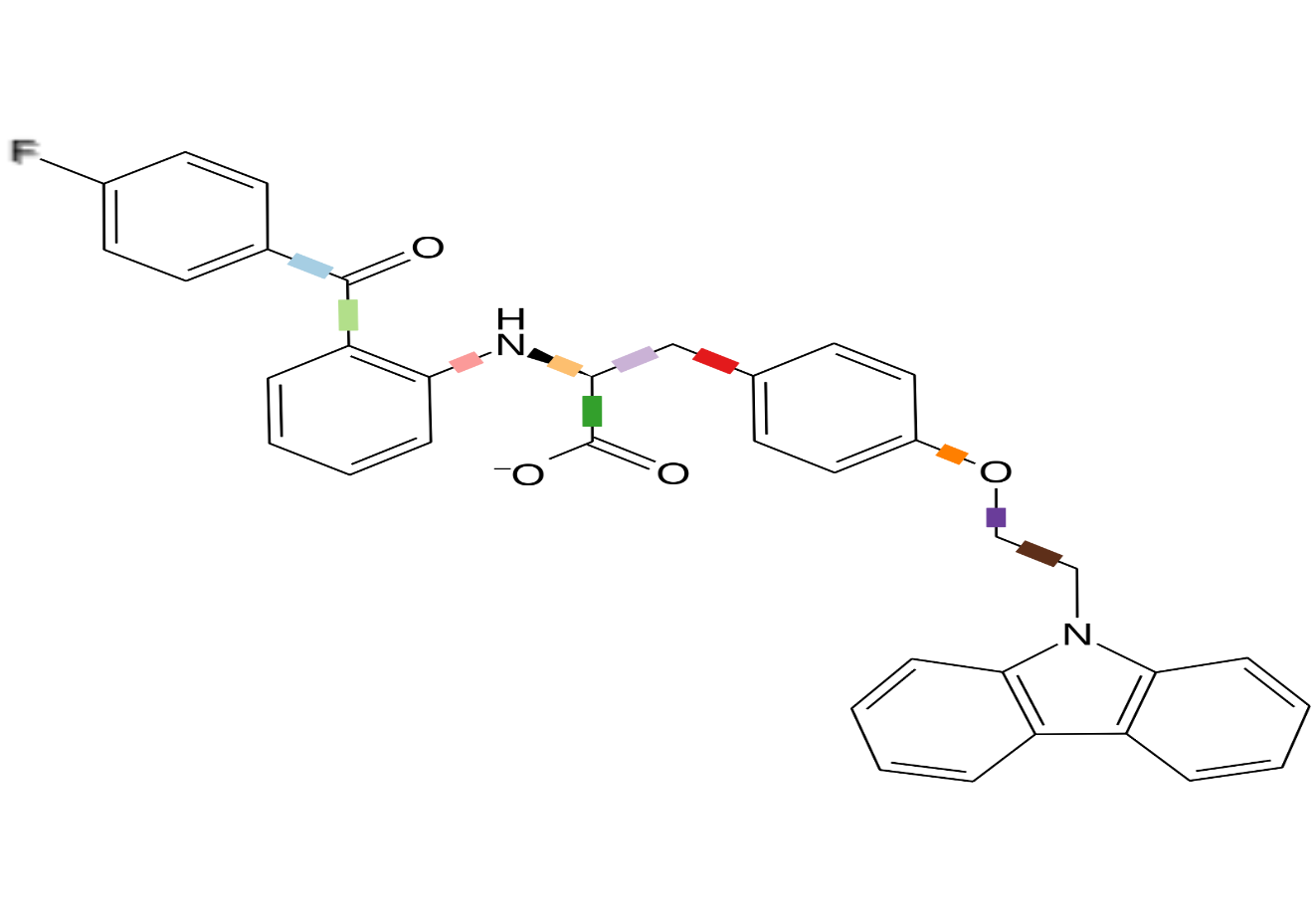 |
| --- |
| 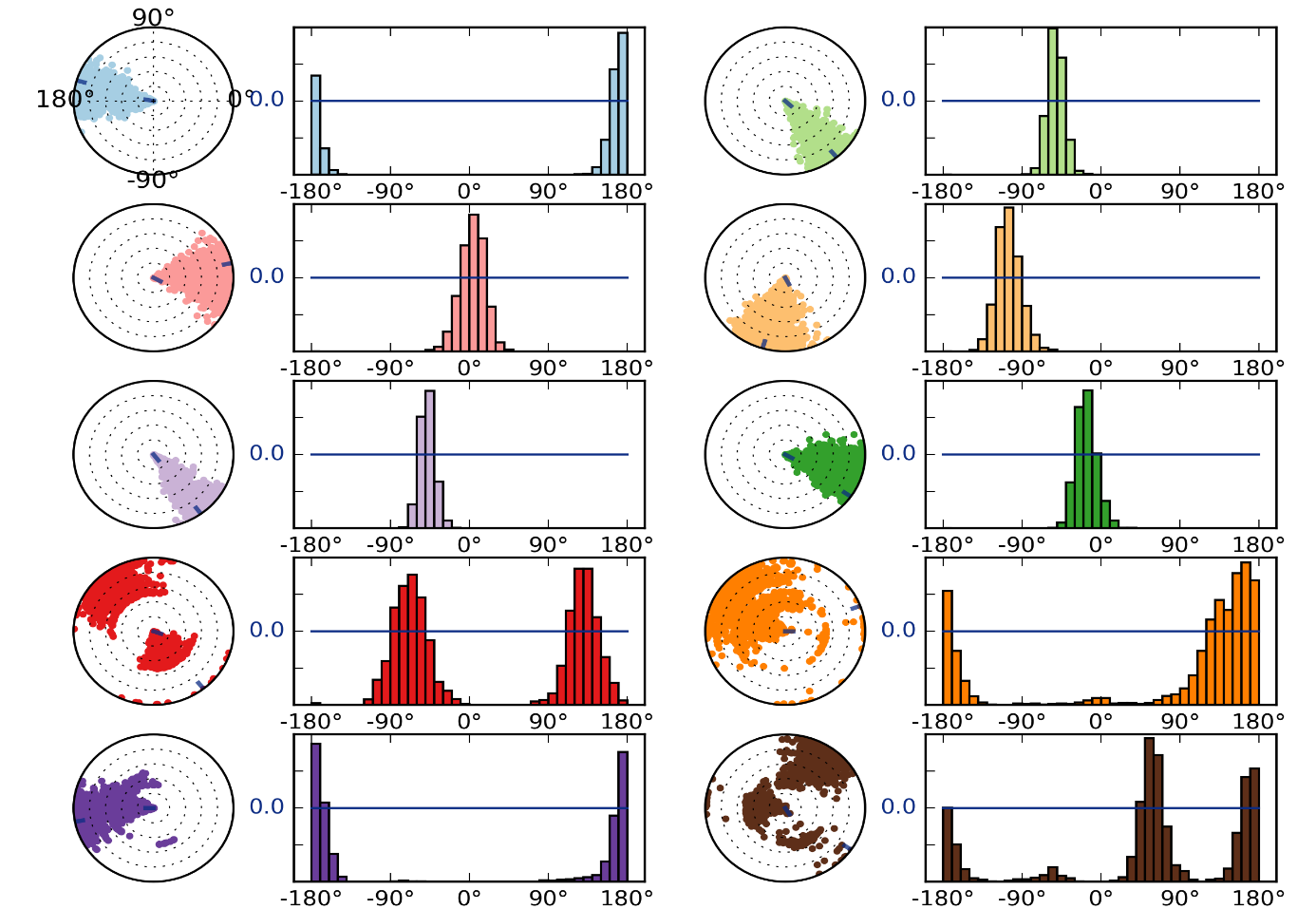  α |
| 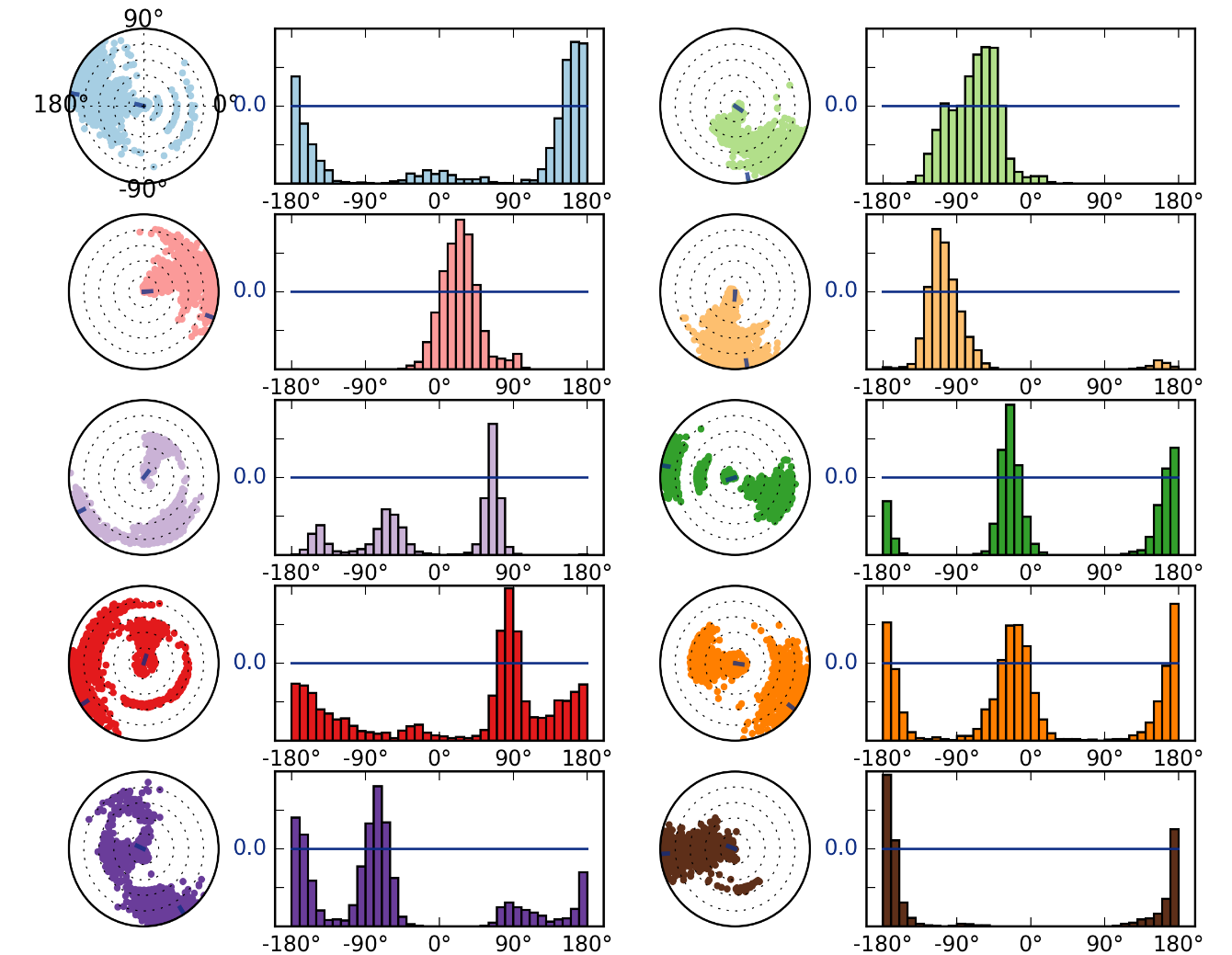  β |
| **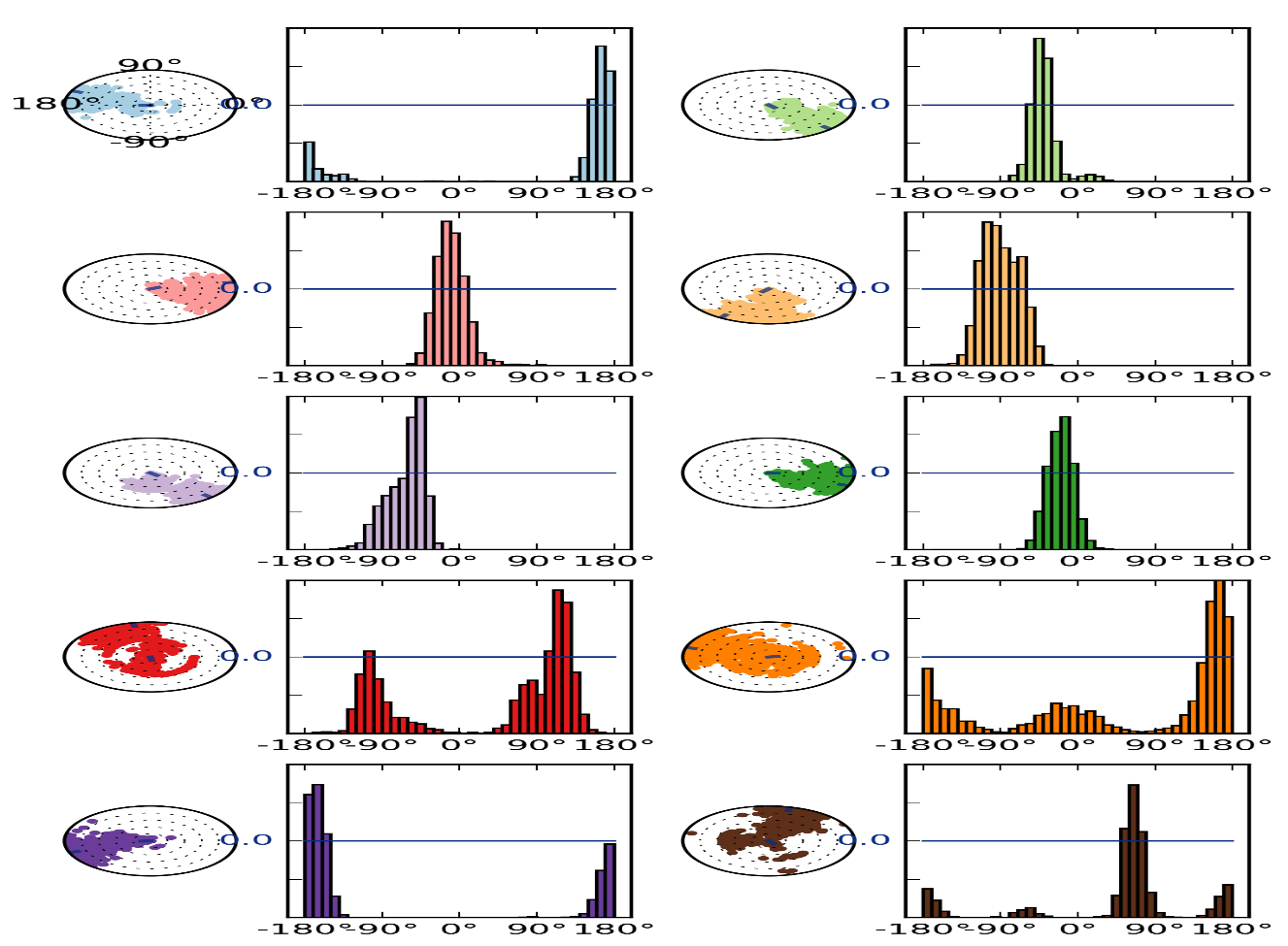**  **γ** |

**Figure S6** Average ligand torsion (dihedral angle) profile of chiglitazar in complex with PPARα, PPARβ, and PPARγ from the combined trajectory runs.

| **Combined**  **A** | **Block 1** | **Block 2** |
| --- | --- | --- |
| **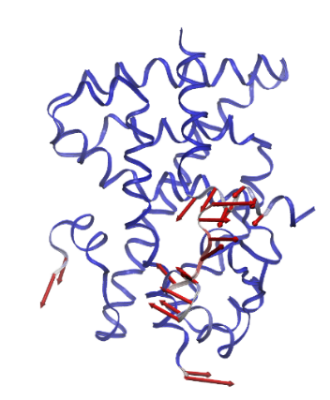**  **1** | **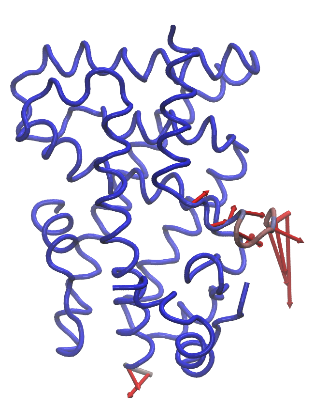** | **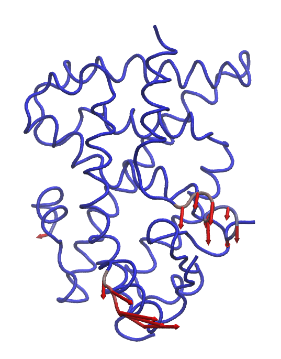** |
| **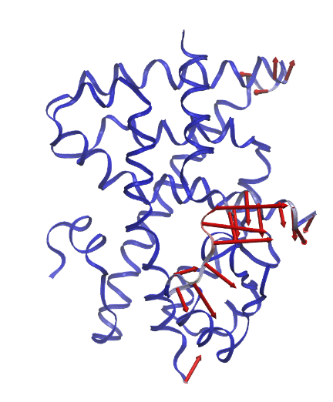**  **2** | **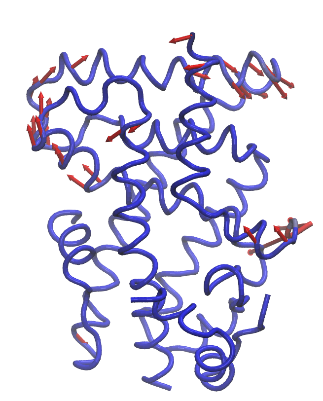** | **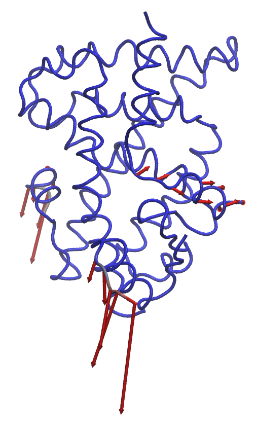** |
| **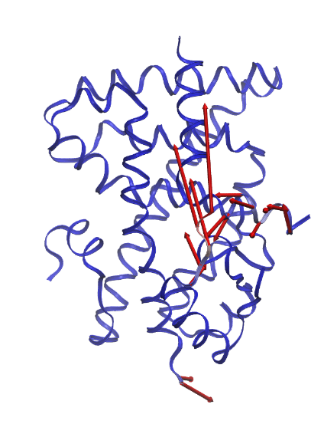**  **3** | **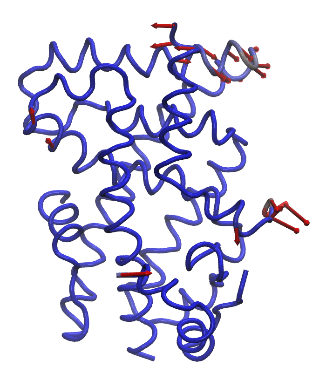** | **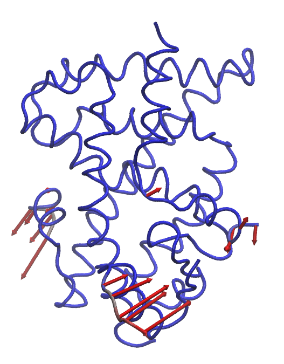** |
| **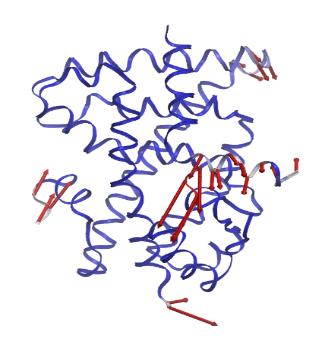**  **4** | **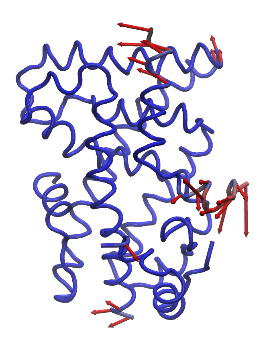** | **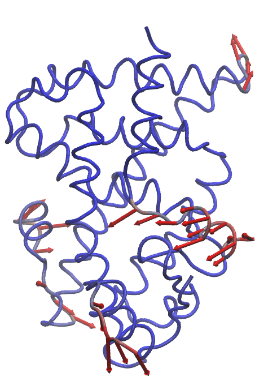** |
| **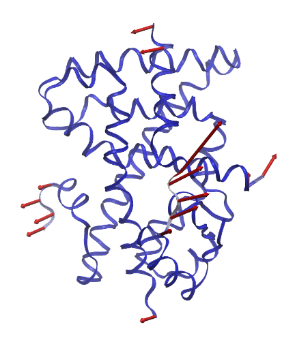**  **5** | **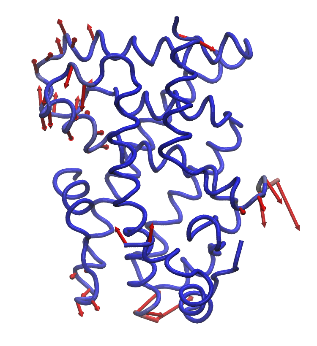** | **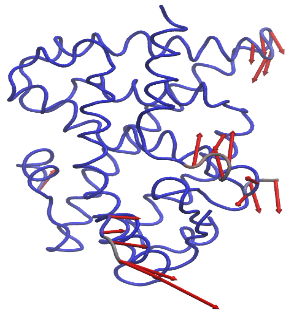** |

| **Combined** | **Block 1** | | **Block 2** | |
| --- | --- | --- | --- | --- |
| **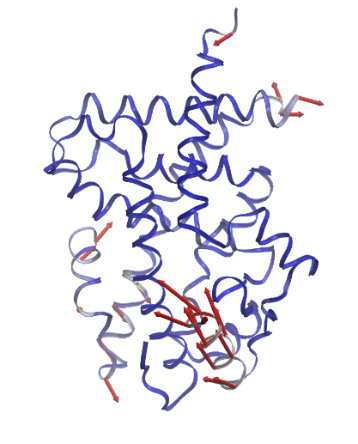**  **1** | **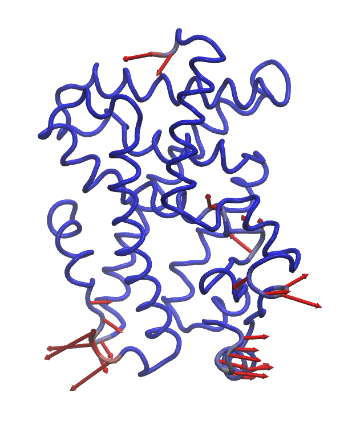** | | **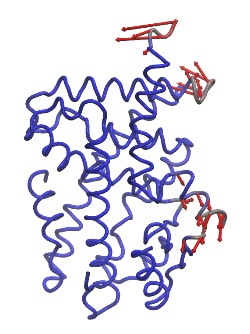** | |
| **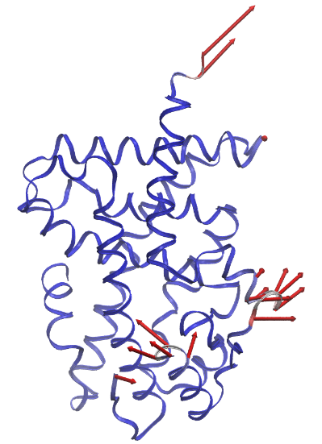**  **2** | **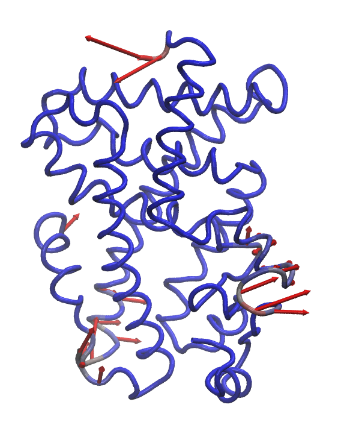** | | **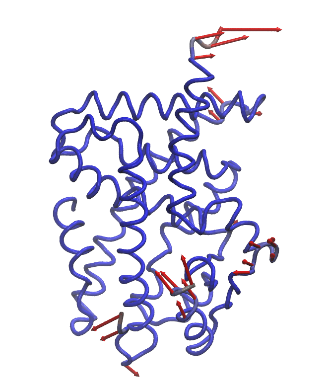** | |
| **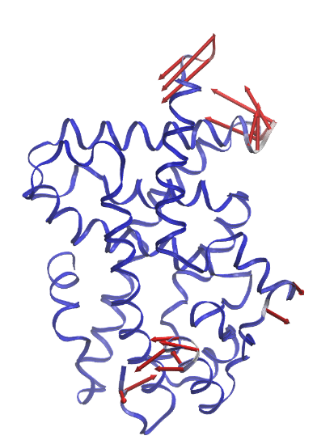**  **3** | **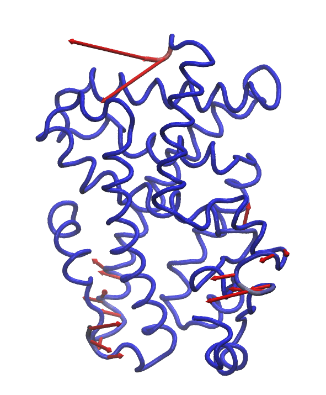** | | **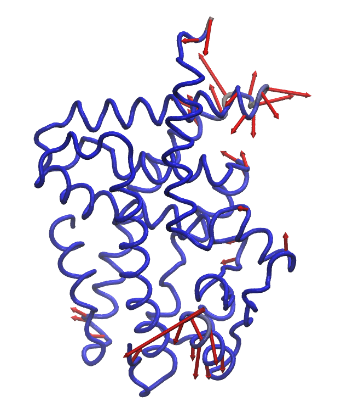** | |
| 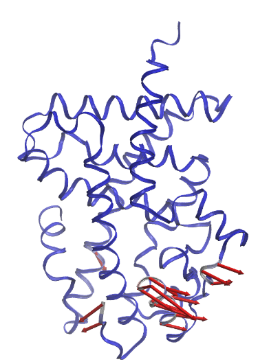  **4** | **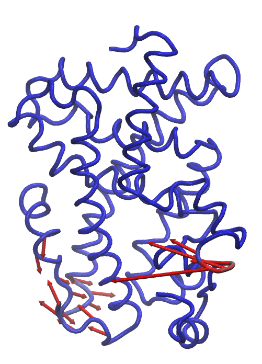** | | **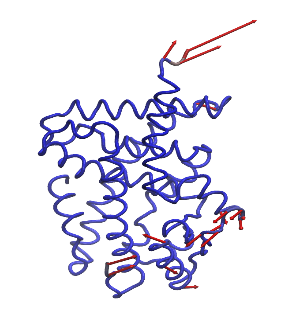** | |
| **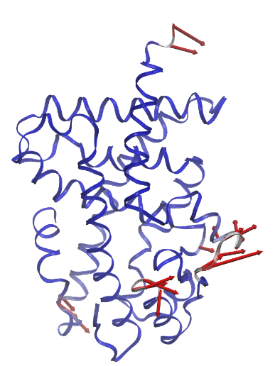**  **5** | **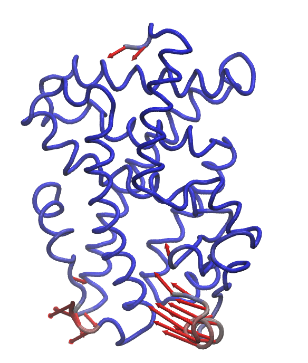** | | **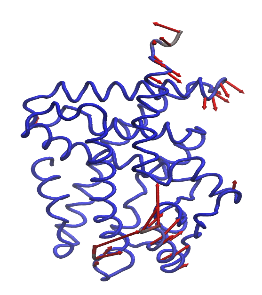** | |
| **Combined**  **B** | | **Block 1** | | **Block 2** |
| **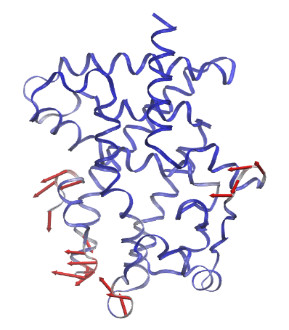**  **C**  **1** | | **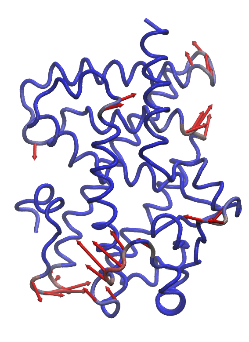** | | **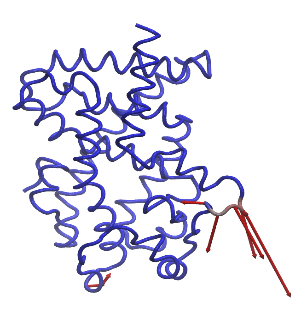** |
| **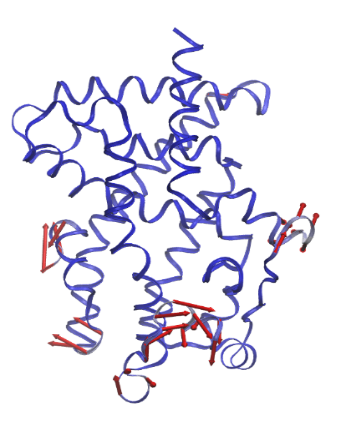**  **2** | | **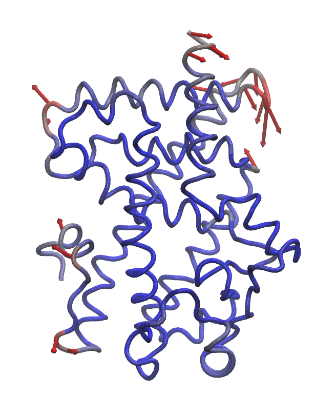** | | **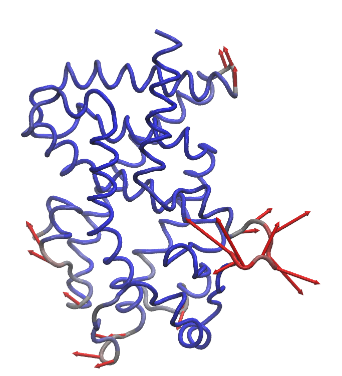** |
| **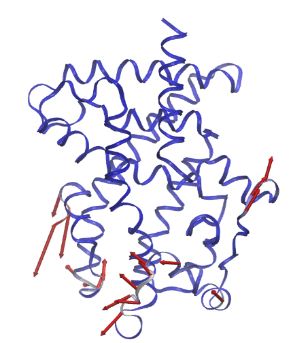**  **3** | | **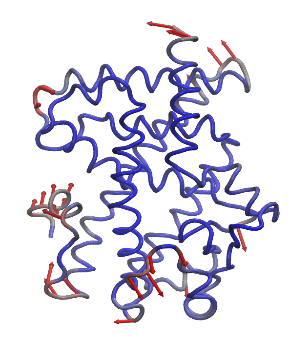** | | **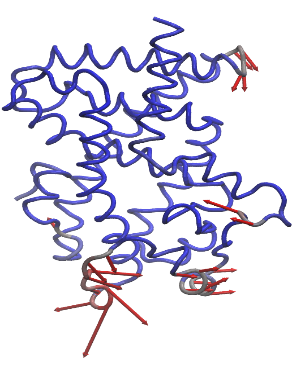** |
| **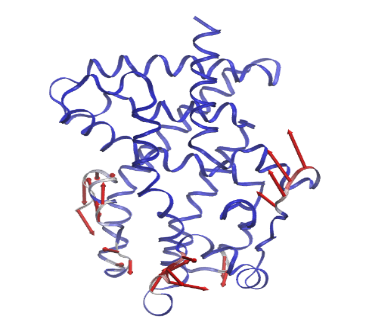**  **4** | | **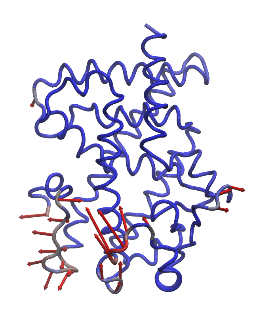** | | **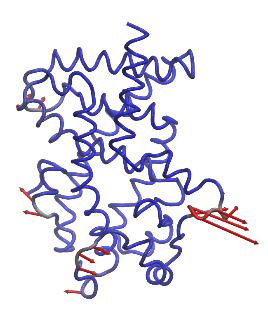** |
| **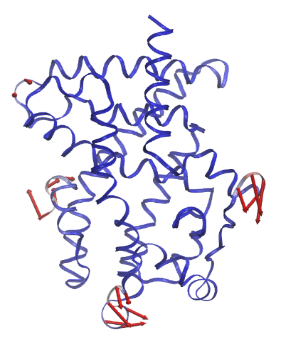**  **5** | | **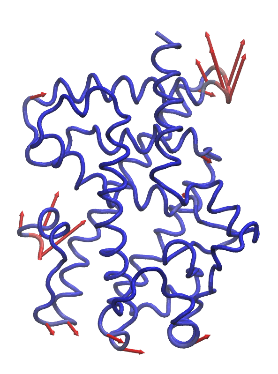** | | **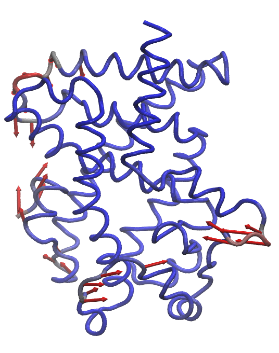** |

**Figure S7.** The top five modes (1-5) of the trajectory based principal component analysis performed using VMD’s normal mode wizard for the combined and blocks of the trajectories for PPARα (A), PPARβ (B), and PPARγ (C). The color scheme is as follows: blue- low movement, grey- moderate movement, red-maximum movement. Vectors of 3.5 Å or greater are shown and represent the directionality of movement where larger vectors represent greater fluctuations.

**Figure S8.** RMSF of the top 5 normal modes of the trajectories, derived from VMD’s normal mode Wizard.

**
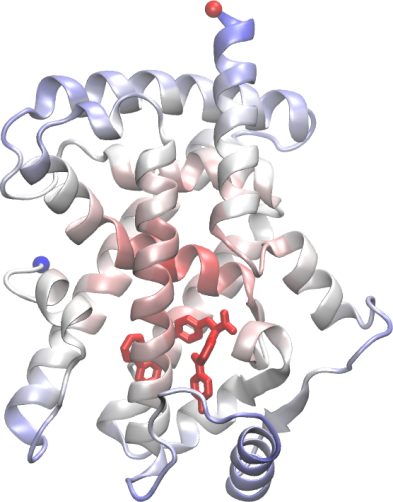
**
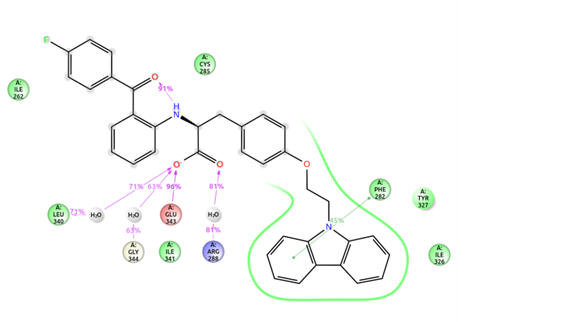


**A**

**B**

**Figure S9.** Docking Pose (A) and 2D Interaction Diagram (B) of Chiglitazar in complex with PPARγ (from PDB ID: 2PRG).

**A**


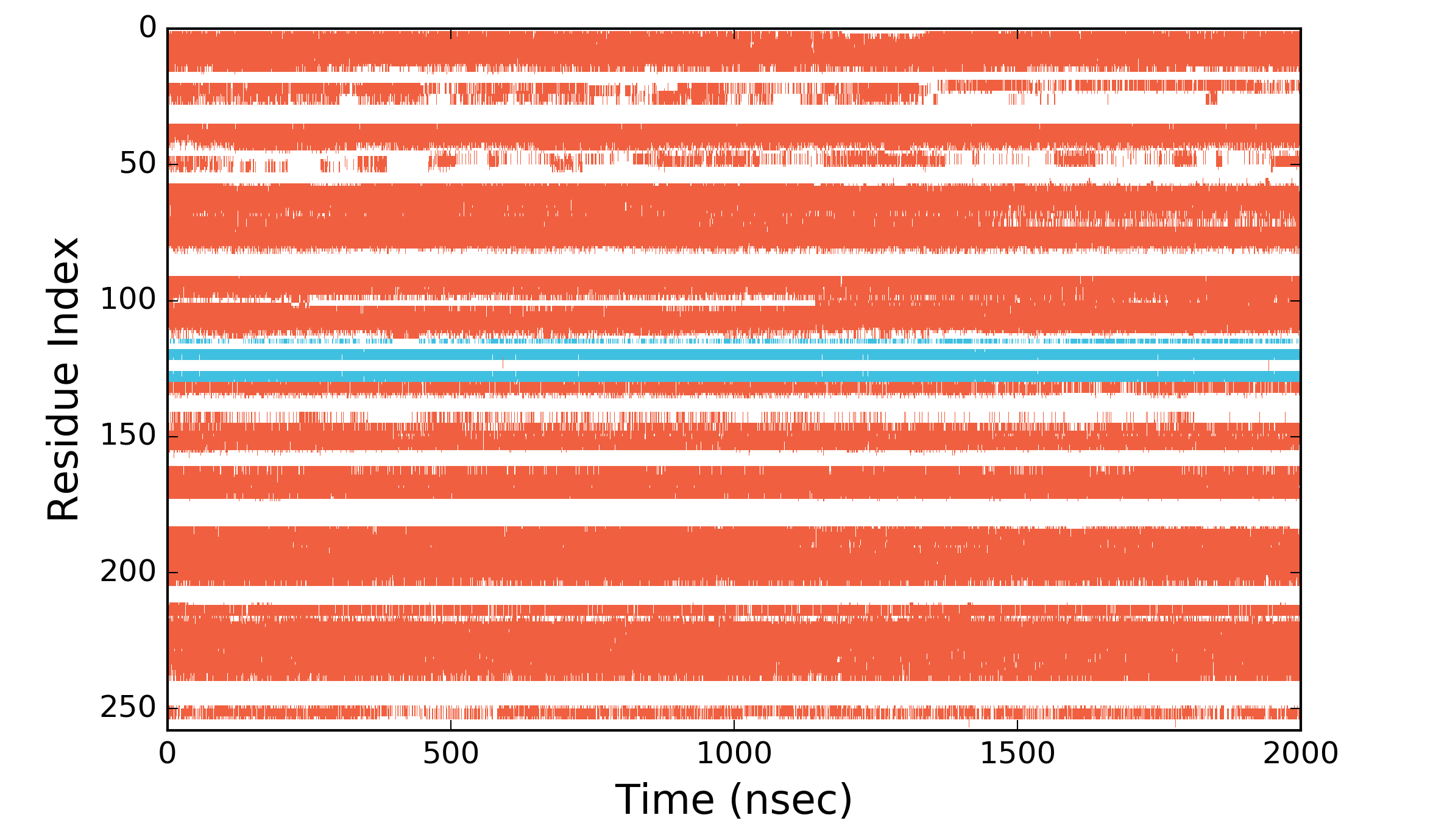

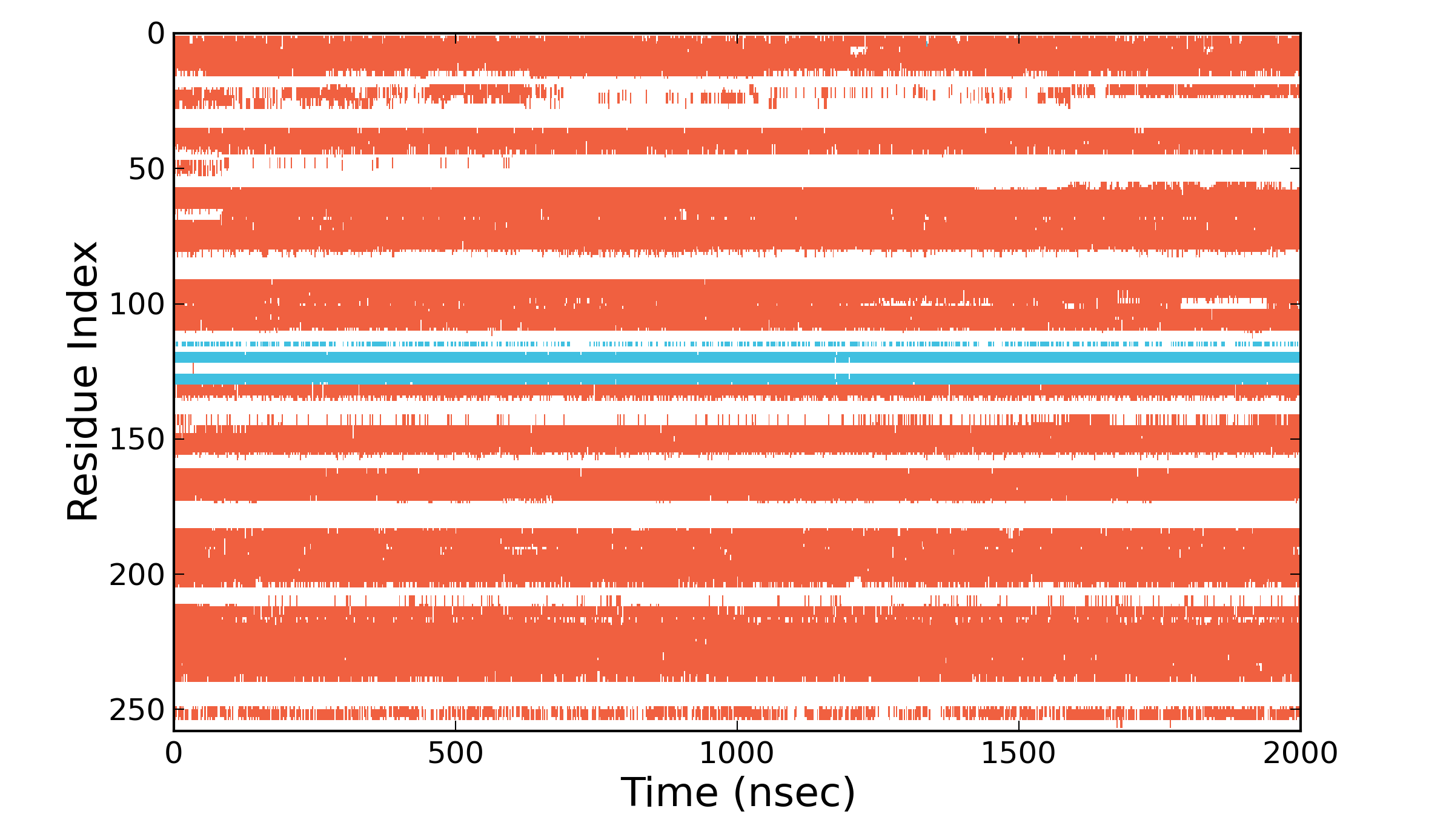


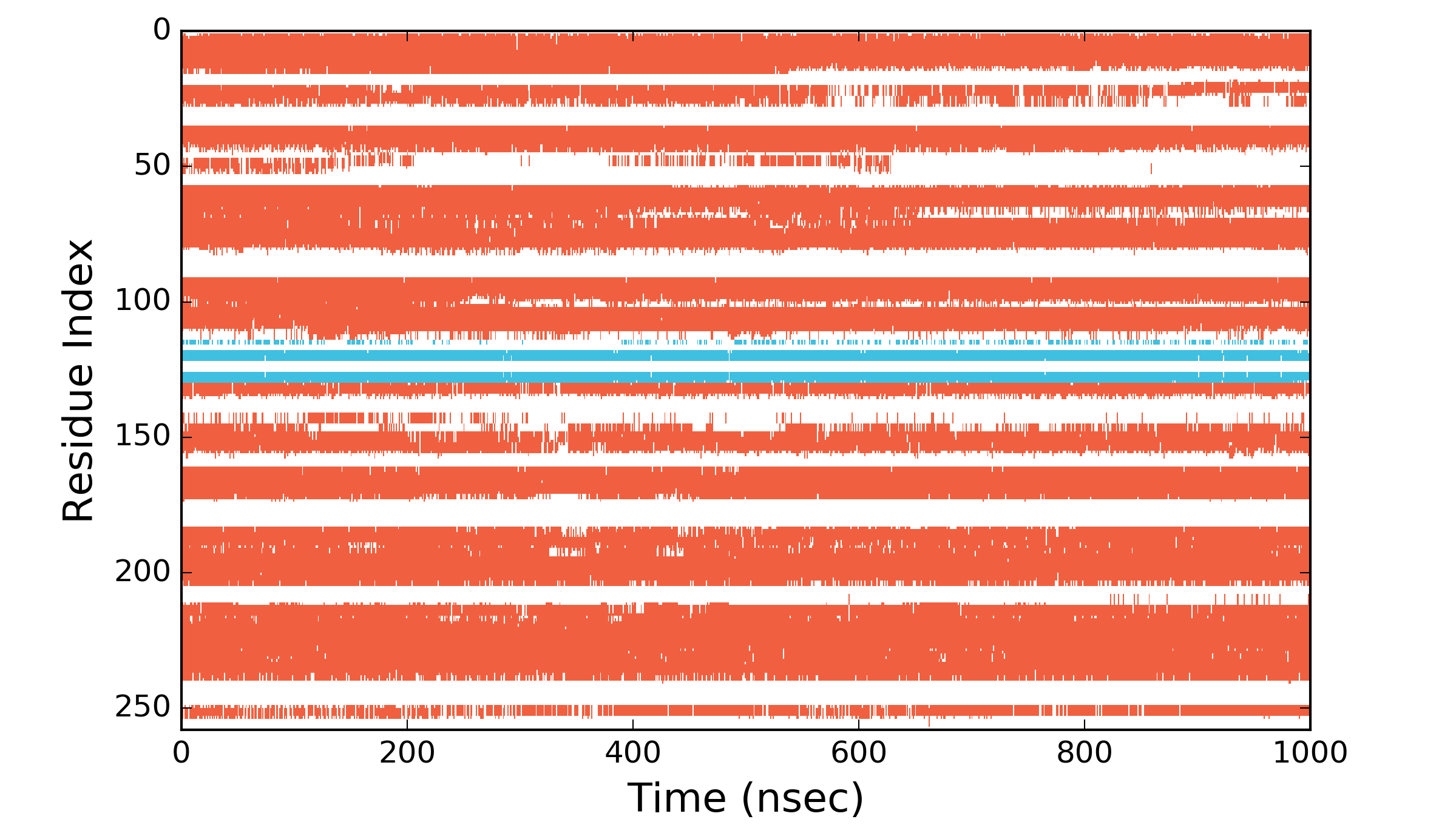


**B**


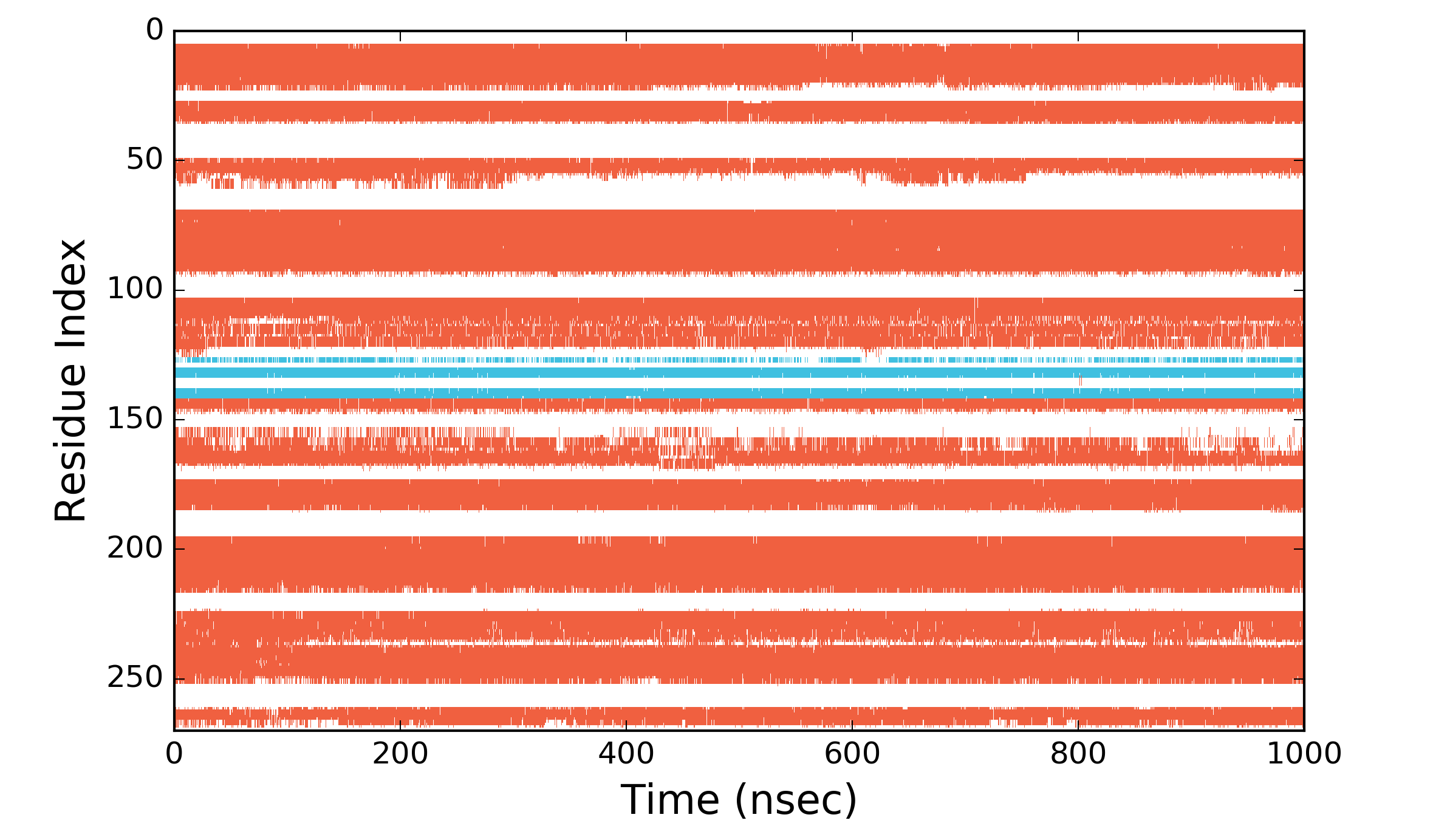

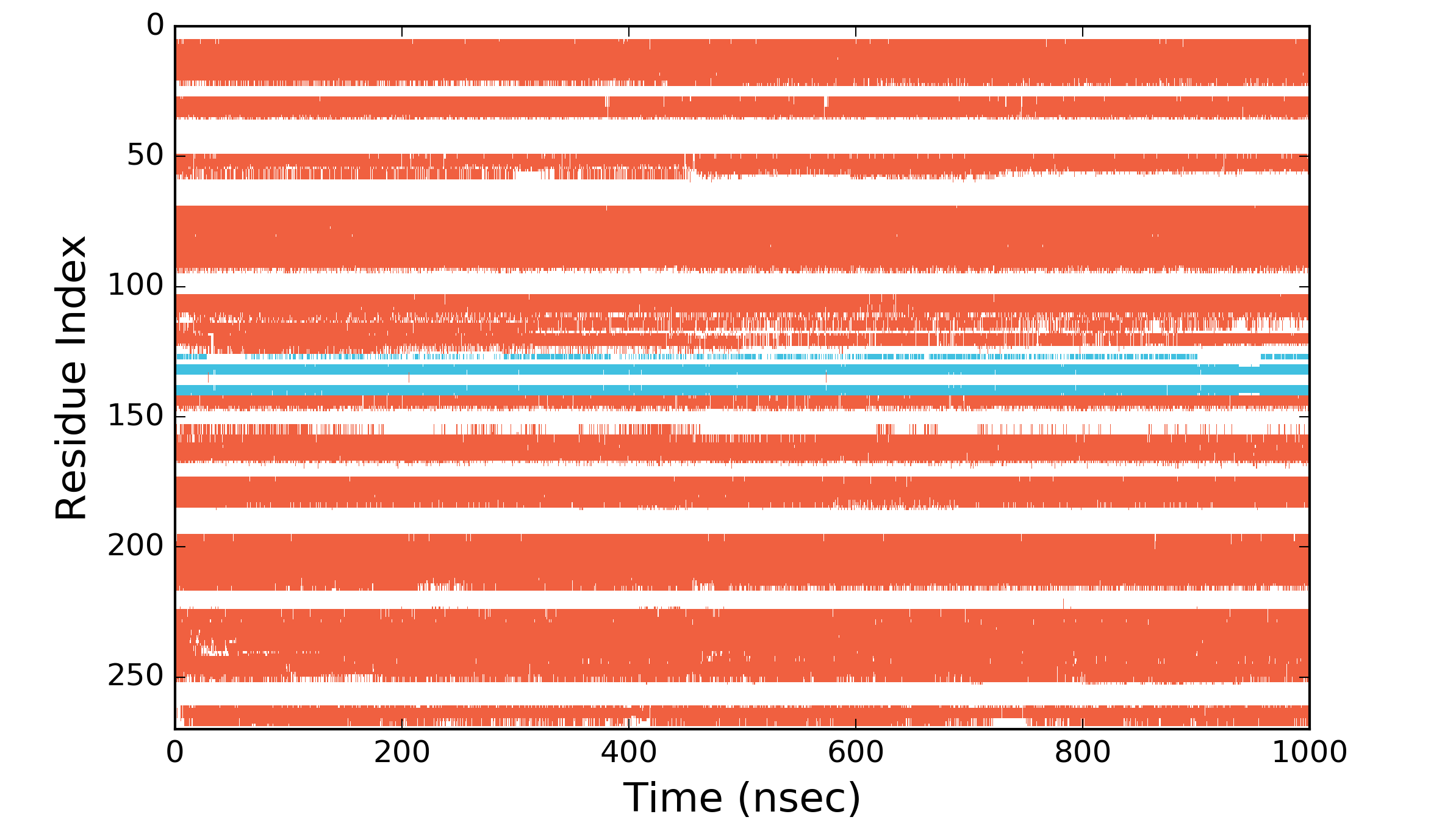

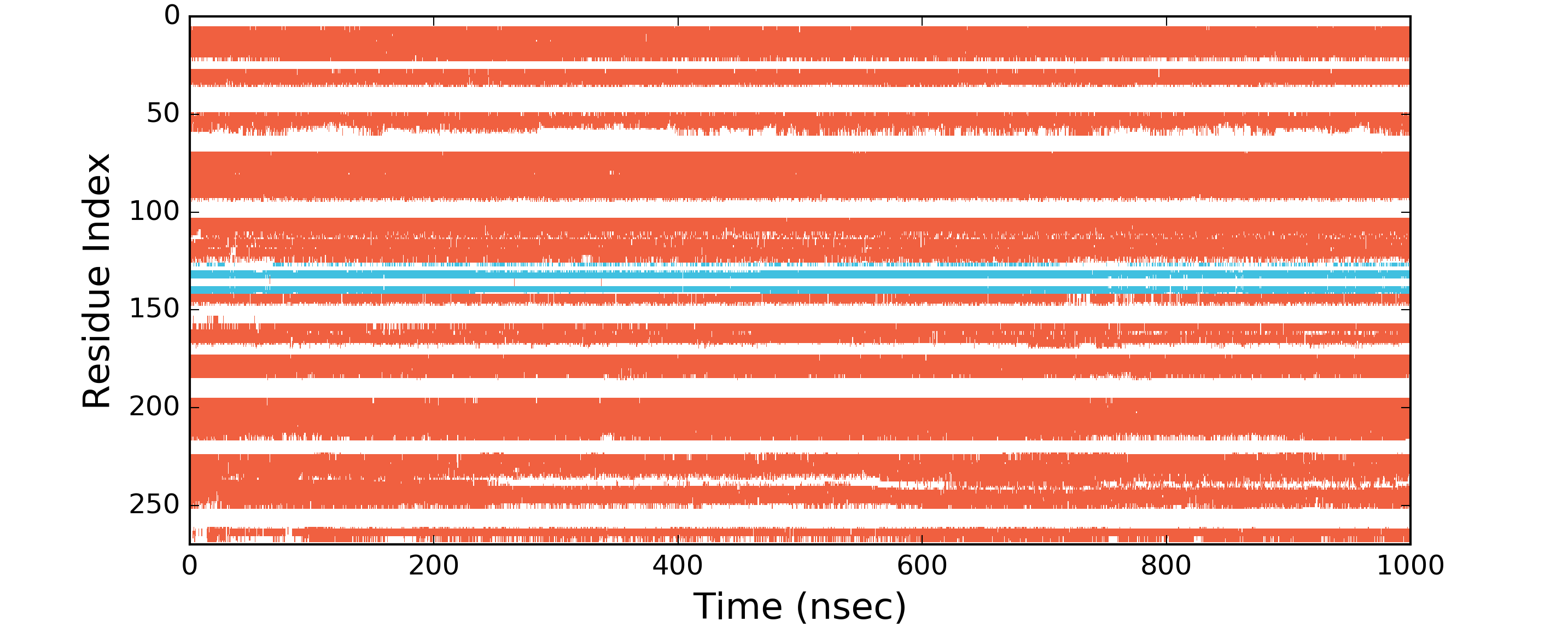


**C**


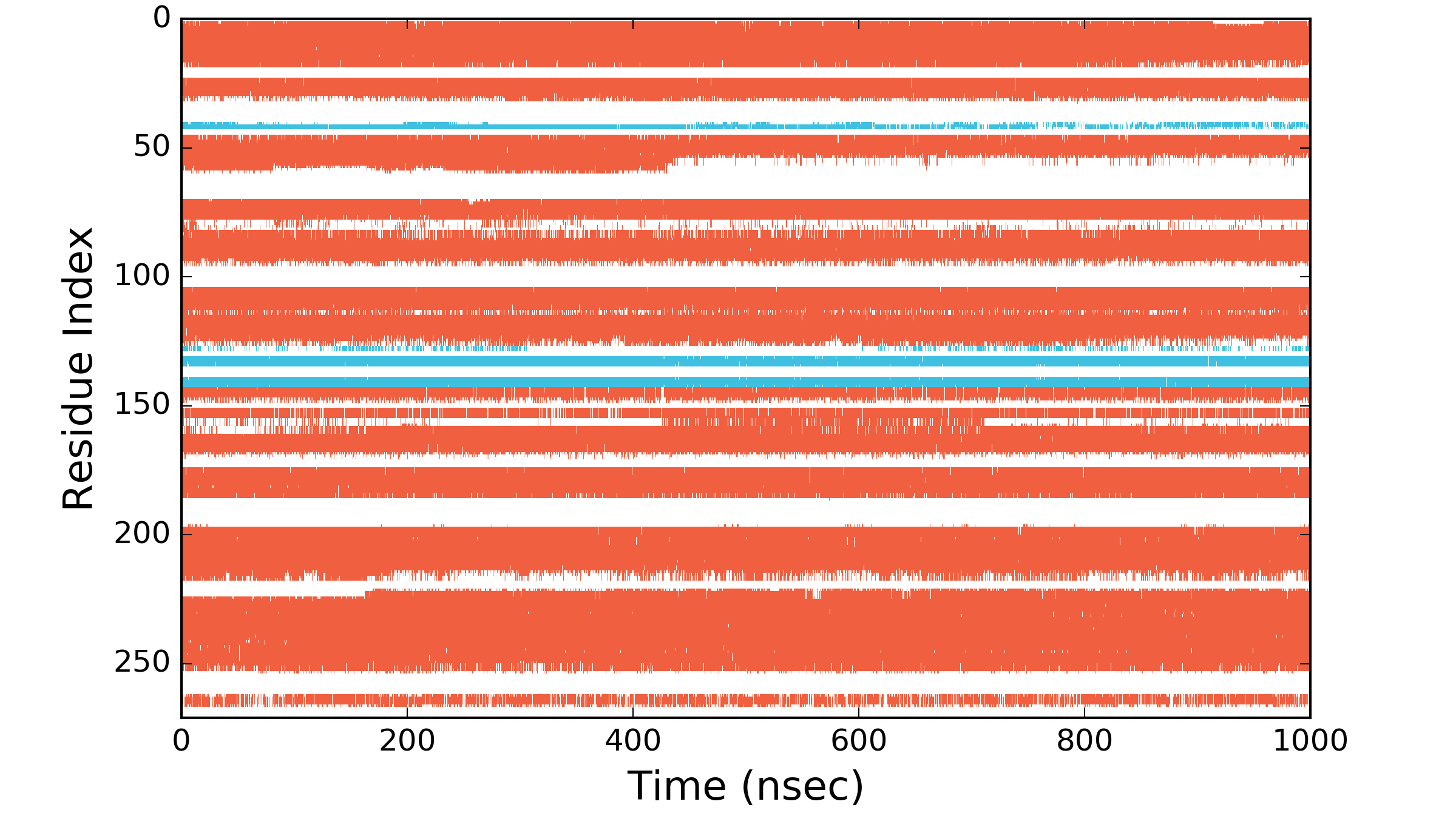

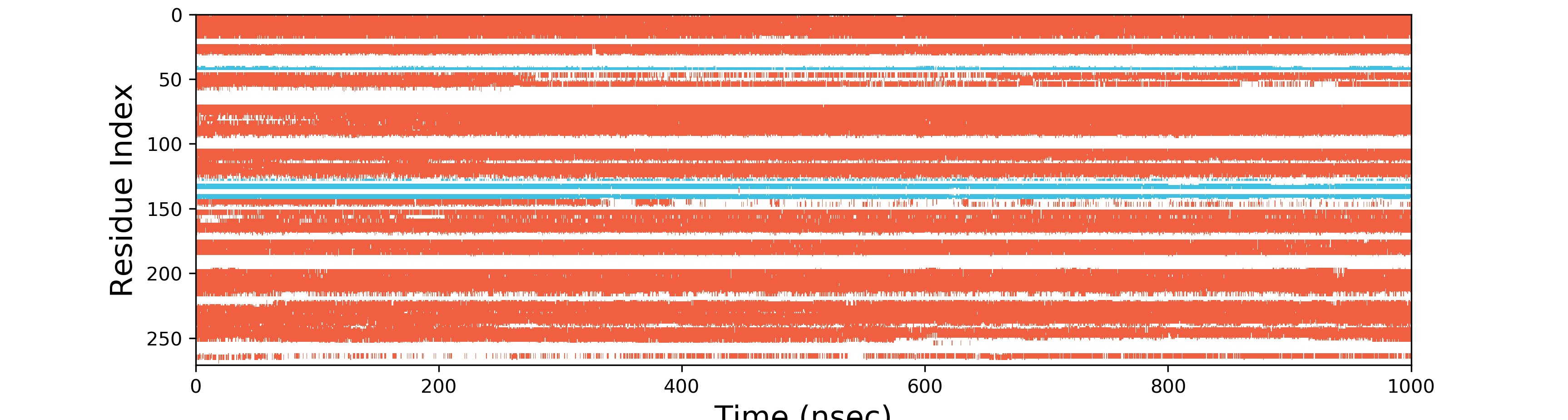

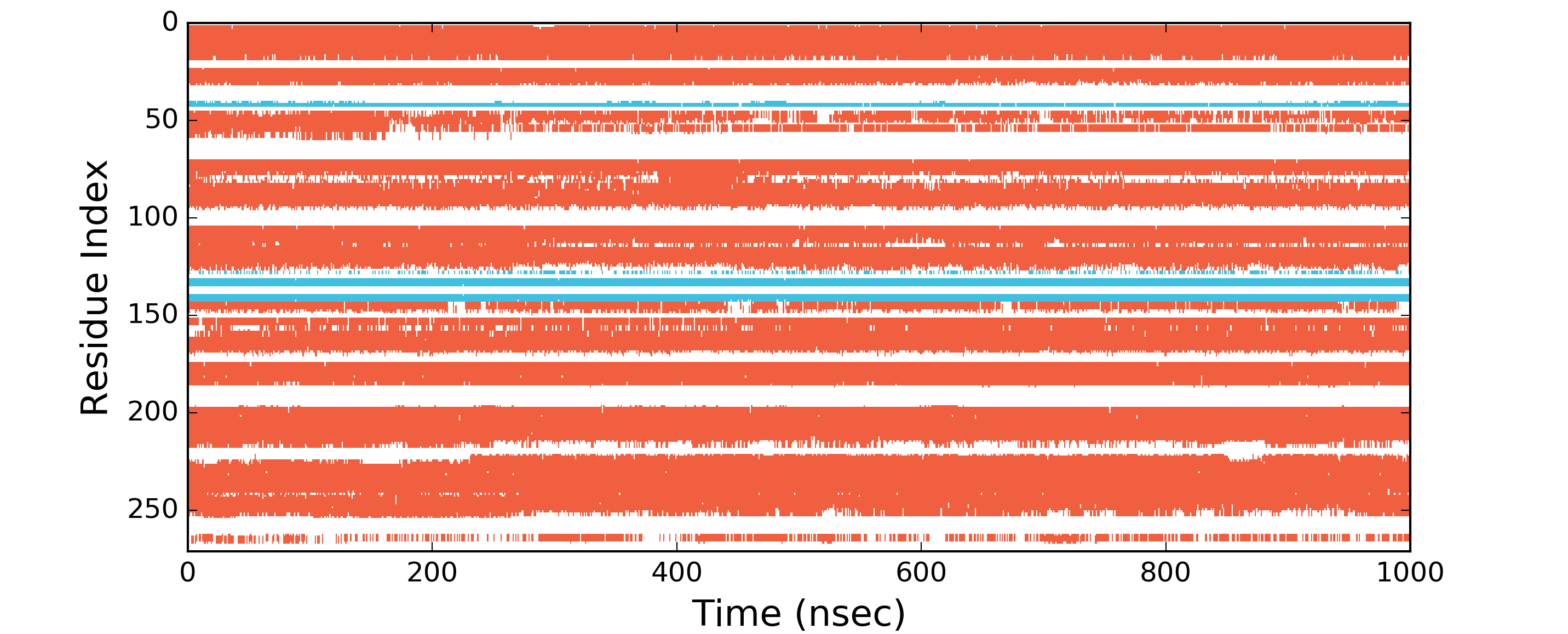


**Figure S10.** Secondary structure element timelines for each of the three trajectories of PPARα (A), PPARβ (B), and PPARγ (C).

| **A** 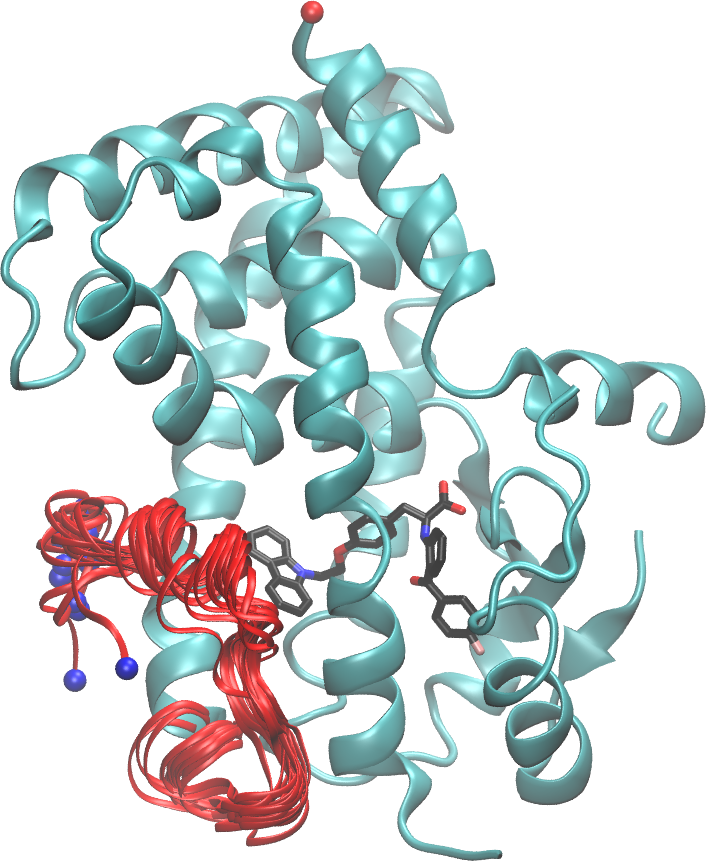 | **B** 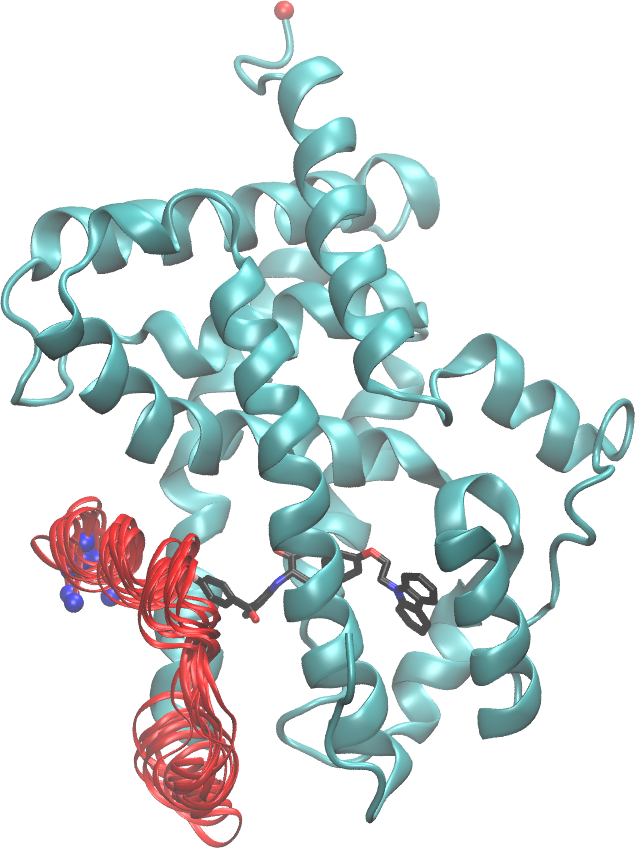 | **C** 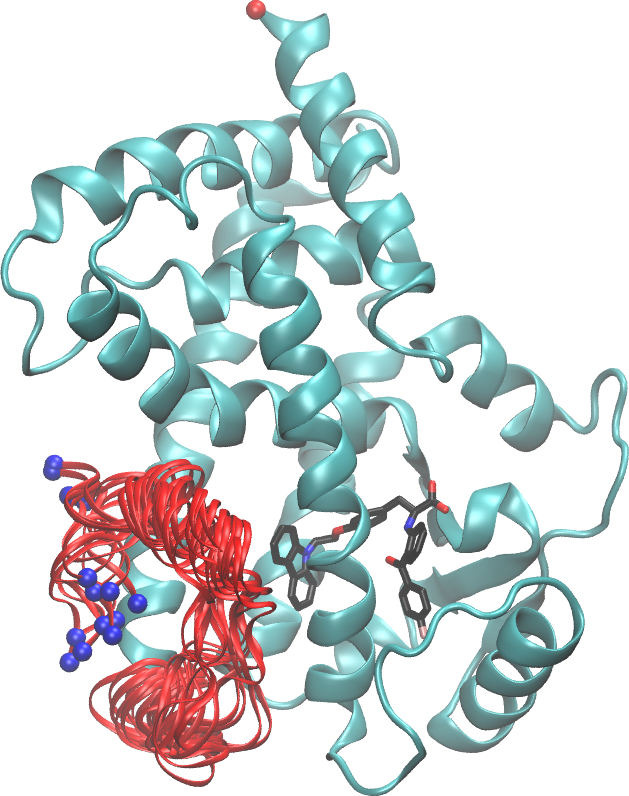 |
| --- | --- | --- |
| 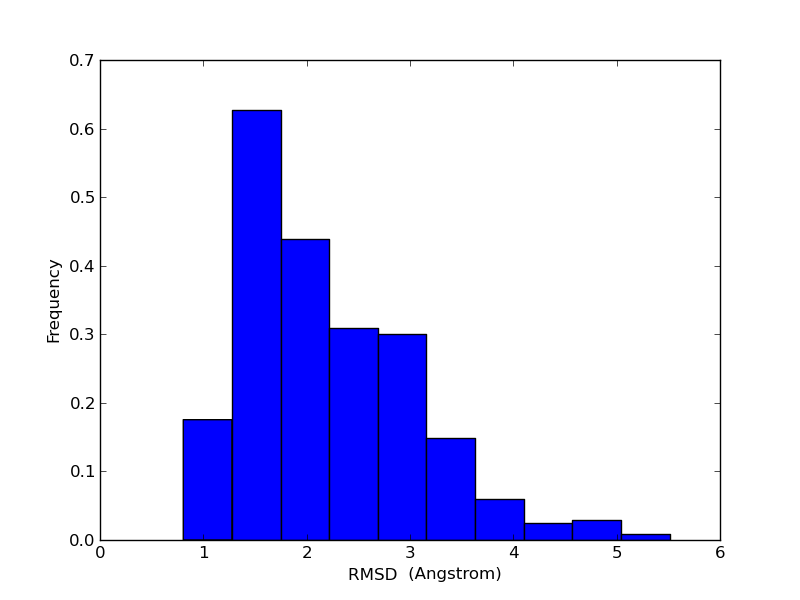 | 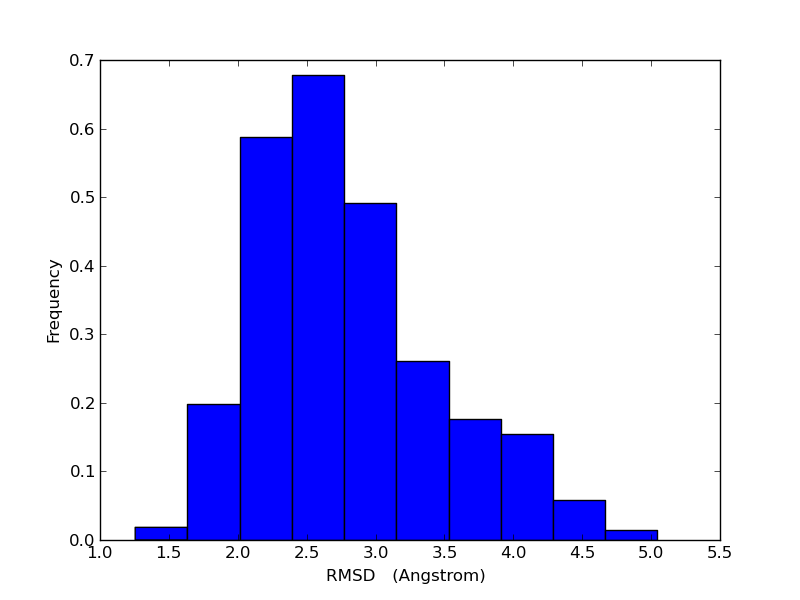 | 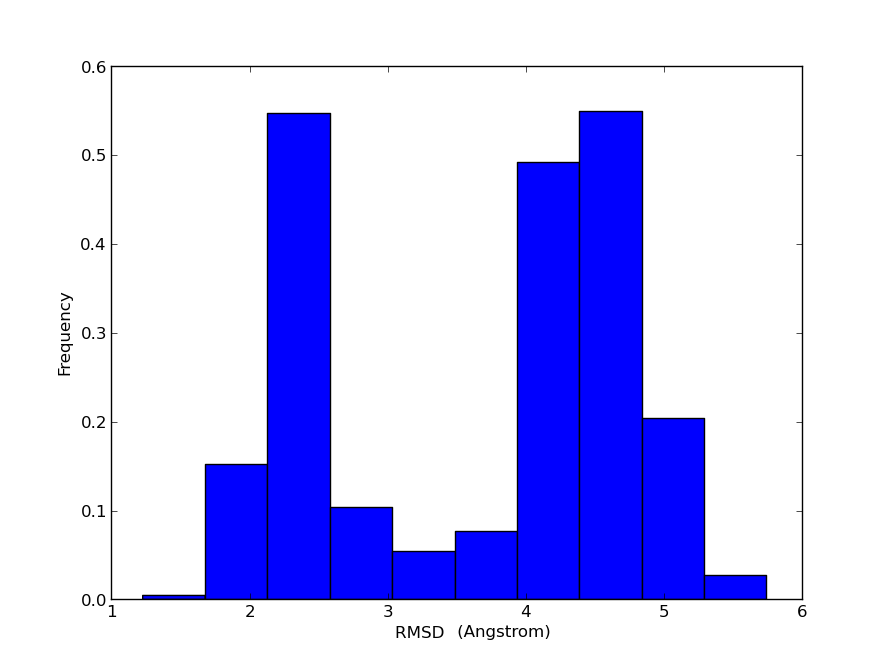 |
|  |  |  |

**Figure S11**. Position of helix 12 (red) over the course of the combined trajectory including a histogram showing the RMSD distribution of helix 12 as well as the time series of the helix 12 RMSD for each trajectory (trajectory 1 blue, trajectory 2 red, trajectory 3 green) for PPARα (A), PPARβ (B), and PPARγ (C). C-terminal represented as a blue ball.

| 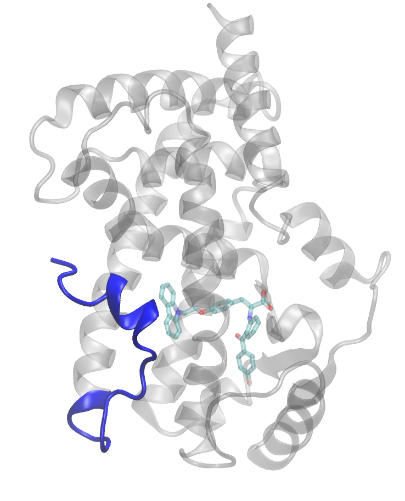 | 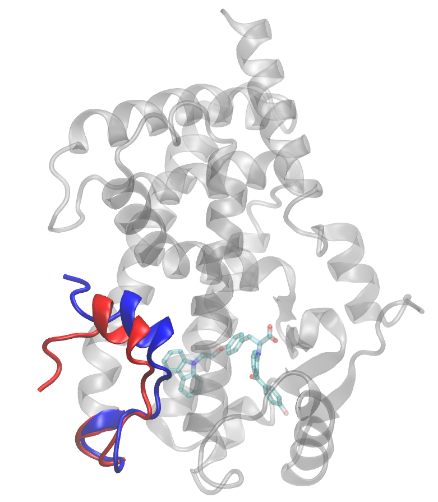 | 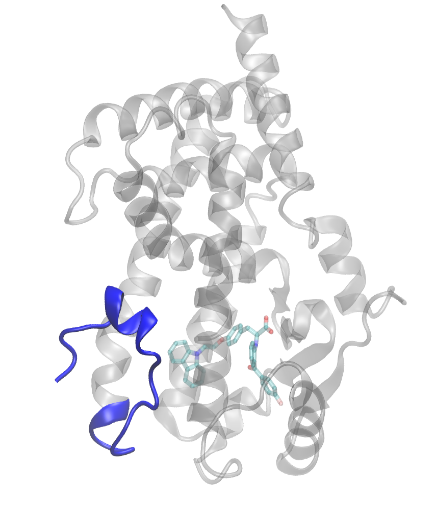 |
| --- | --- | --- |
| RMSD: 2.5 Å | Superimposition | RMSD: 4.5 Å |

Figure S12. Two most abundant conformations of helix 12 based on RMSD. Superimposition shows conformation of helix 12 at 2.5 Å RMSD in blue and at 4.5 Å RMSD in red.
